# Synergistic piezo-immunotherapy enabled by lithium-doped SrTiO_3_ nanocatalysts for potent tumor ablation through ROS generation and immune activation

**DOI:** 10.1093/rb/rbag030

**Published:** 2026-03-24

**Authors:** Yuzheng Gao, Yichun Zhang, Shuyan Zhang, Zhiwei Yang, Wenjing Liu, Jing Zhang, Zhipeng Gu, Xianchun Chen

**Affiliations:** College of Polymer Science and Engineering, Sichuan University, No.24 South Section 1, Yihuan Road, Chengdu 610065, China; College of Polymer Science and Engineering, Sichuan University, No.24 South Section 1, Yihuan Road, Chengdu 610065, China; College of Polymer Science and Engineering, Sichuan University, No.24 South Section 1, Yihuan Road, Chengdu 610065, China; College of Architecture and Environment, Sichuan University, No.24 South Section 1, Yihuan Road, Chengdu 610065, China; College of Polymer Science and Engineering, Sichuan University, No.24 South Section 1, Yihuan Road, Chengdu 610065, China; College of Architecture and Environment, Sichuan University, No.24 South Section 1, Yihuan Road, Chengdu 610065, China; College of Polymer Science and Engineering, Sichuan University, No.24 South Section 1, Yihuan Road, Chengdu 610065, China; College of Polymer Science and Engineering, Sichuan University, No.24 South Section 1, Yihuan Road, Chengdu 610065, China

**Keywords:** piezocatalysis, antitumor therapy, elemental doping, oxygen vacancies, synergistic immunotherapy

## Abstract

Piezocatalytic tumor therapy represents an emerging approach in cancer treatment, leveraging sonosensitizers to generate reactive oxygen species (ROS) under ultrasound (US) irradiation for effective tumor eradication. However, enhancing ROS production efficiency remains a critical challenge in this field. In this study, SrTiO_3_ (STO) was selected as the base piezocatalytic material, and its performance was optimized through a combined strategy of lithium doping and oxygen vacancy engineering. The modified material (designated 1.5LSTO) exhibits substantially enhanced local electrical responses. As quantified by PFM, its surface potential and piezoelectric (butterfly-type) amplitude were ∼2.23-fold higher than those of the unmodified sample. The optimally modified material, designated as 1.5LSTO, exhibited a 1.44-fold enhancement in piezocatalytic activity compared to pristine STO under US exposure, enabling efficient generation of hydroxyl radicals (•OH) and superoxide anions (•$O_{2}^{\;-}$). *In vitro* experiments demonstrated significant cytotoxicity of 1.5LSTO against tumor cells. Furthermore, *in vivo* studies using an intestinal tumor-bearing mouse model confirmed that US-activated 1.5LSTO effectively suppressed tumor proliferation and promoted apoptosis. Notably, lithium doping was found to significantly upregulate CD8^+^ T cell expression, indicating an immunomodulatory effect. The integration of piezocatalysis with immune activation resulted in a multimodal synergistic therapy that substantially improved overall antitumor efficacy. This work provides an innovative material-based strategy for enhancing tumor treatment through functional modulation and synergistic mechanisms.

## Graphical abstract

**Figure:**
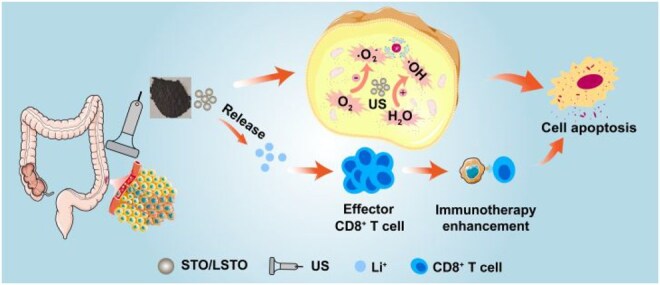

## Introduction

Colorectal cancer ranks as the third most commonly diagnosed malignancy worldwide [1]. Current standard treatments—including surgery, chemotherapy and radiotherapy—are often compromised by systemic toxicity, therapy resistance and tumor recurrence [2, 3]. Meanwhile, emerging therapies such as immunotherapy and cell therapy face limitations due to high costs and considerable inter-patient variability [4]. In light of these challenges, sonodynamic therapy (SDT) has emerged as a promising noninvasive alternative, offering deep tissue penetration, precise spatiotemporal control and relatively low treatment costs [5–8].

The fundamental mechanism of SDT relies on the US-triggered activation of piezoelectric sonosensitizers, which enables *in situ* generation of cytotoxic reactive oxygen species (ROS) at tumor sites, leading to targeted tumor cell destruction [9, 10]. Central to this process are nanocatalytic systems, which play an indispensable role in mediating and amplifying SDT outcomes [11–13]. By rational design of such systems, it is possible to enhance tumor accumulation and cellular internalization of sonosensitizers, while simultaneously augmenting ROS generation and disrupting tumor defense mechanisms. These integrated strategies collectively contribute to significantly improving the therapeutic performance of SDT.

Strontium titanate (SrTiO_3_) is a perovskite-type material with pronounced piezoelectric catalytic activity [14, 15]. Intriguingly, beyond its physical functionalities, the constituent strontium (Sr) ions exhibit a unique biological capacity to suppress tumor growth and metastasis, positioning SrTiO_3_ as a promising sonosensitizer for SDT [16, 17]. Nevertheless, the low charge carrier density and rapid carrier recombination of SrTiO_3_ limit its ability of ROS generation, thereby reducing its therapeutic efficacy. To address these limitations, element doping emerges as a potent strategy. This approach is anticipated to not only enhance charge carrier dynamics but also concurrently introduce oxygen vacancies (*V*_0_) and modulate the electronic structure of the host material, as dopants can act as electron donors [18–21]. Compared to other methods, its principal advantage lies in its ability to orchestrate multifaceted structural and electronic modifications, overcoming the constraints of single-parameter optimization and enabling synergistic performance enhancement.

Among various dopants, lithium (Li) is particularly attractive due to its small ionic radius, which facilitates effective incorporation into the crystal lattice and is predicted to augment the material’s polarization and piezoelectric response [22, 23]. Moreover, Li itself possesses documented biological effects, including antioxidant, anti-inflammatory and notably, immunomodulatory activities. Specifically, lithium has demonstrated potential in enhancing T-cell function, suggesting a promising avenue for bolstering immune-mediated antitumor responses [24–26]. Capitalizing on these dual attributes of lithium, we postulate that Li doping can significantly optimize the piezocatalytic performance of SrTiO_3_, thereby advancing the frontiers of piezocatalytic tumor therapy [27].

In this study, oxygen-deficient lithium-doped SrTiO_3-x_ (LSTO) nanocatalysts were synthesized via a precursor route using black lithium titanate (LTO) to overcome the limitations of conventional piezoelectric materials. Lithium doping was demonstrated to concurrently elevate the charge carrier density and introduce oxygen vacancies, which synergistically enhanced the piezocatalytic activity. And 1.5LSTO was optimized out for its strongest ROS generation ability under US. Biological evaluations, both *in vitro* and *in vivo*, confirmed that these nanoparticles effectively ablated tumor cells and tissues upon US excitation while maintaining high biocompatibility under physiological conditions. Furthermore, lithium doping was found to significantly promote the infiltration of CD8^+^ T cells into the tumor microenvironment, enabling a combined therapeutic outcome that integrates piezocatalysis with immunotherapy and substantially augments antitumor efficacy. Collectively, these findings underscore the potential of 1.5LSTO as an efficient and cost-effective sonosensitizer for advanced tumor treatment, paving a new avenue for piezoelectric catalysis-based therapeutic strategies.

## Experiment section

### Synthesis of lithium-doped strontium titanate

The synthesis of LSTO involved two sequential steps as follows:

#### Preparation of lithium-reduced TiO_2_ precursors (Li-TiO_2_)

All operations involving metallic lithium were carried out in an argon-filled glovebox to avoid oxidation. Titanium dioxide powder (0.025 mol) was precisely weighed. Lithium foil was then cut into small pieces and added to the TiO_2_ powder in molar ratios (Li: TiO_2_) of 0.5:1, 1.0:1, 1.5:1 and 2.0:1, corresponding to Li quantities of 0.0125, 0.025, 0.0375 and 0.05 mol, respectively. The mixture was ground thoroughly in an agate mortar using a pestle, with periodic homogenization using a spatula, until a uniform blend was achieved. During this grinding process, the mixture spontaneously ignited. The resulting black combustion products were collected and washed with 0.2 M acetic acid to remove soluble lithium salts, followed by thorough rinsing with deionized water and anhydrous ethanol. The final precursor powders, corresponding to their respective Li: TiO_2_ molar ratios, were labeled as 0.5LTO, 1.0LTO, 1.5LTO and 2.0LTO, and the precursor 1.5LTO was optimized out for LSTO synthesis.

#### Hydrothermal synthesis of LSTO

A hydrothermal synthesis was performed as follows: 37.5 mL of deionized water was placed into a Teflon-lined reactor vessel. Subsequently, 6.00 g of sodium hydroxide (NaOH) were added to the solution and stirred until completely dissolved. Then, 0.75 g of 1.5LTO was added to the alkaline solution and stirred for 10 min. Next, 4.97 g of strontium hydroxide octahydrate [Sr(OH)_2_·8H_2_O] were added, and the mixture was stirred vigorously for 1h to ensure homogenization. The sealed reactor was then placed in an oven and maintained at 210°C for 24 h. After cooling, the resulting precipitate was collected, washed repeatedly with 1 M HNO_3_ to remove residual hydroxide ions and soluble species, and rinsed thoroughly with deionized water and anhydrous ethanol. The final lithium-doped strontium titanate powder synthesized using the 1.5LTO precursor is designated 1.5LSTO. A reference strontium titanate sample (STO) was also synthesized under the identical conditions using the pristine TiO_2_ without Li reduction.

### Property analysis and characterization

The crystal structures of the various samples were investigated and confirmed through X-ray diffraction (XRD, XRD-6100, Japan). Surface morphology and microstructure were characterized using scanning electron microscope (SEM, Apreo S, USA). Surface defects and phase evolution were examined using high-resolution transmission electron microscope (HRTEM, Tecnai G2 F20, USA). Elemental composition and chemical states of the samples were analyzed using X-ray photoelectron spectroscopy (XPS, Escalab 250Xi, USA), with external carbon contamination serving as the charging reference, and the 284.80 eV C-C peak used for charge correction. The piezoelectric and ferroelectric properties of the different samples were investigated using piezoresponse force microscopy (PFM, BRUKER Dimension Icon, Germany). Oxygen vacancies were detected under ambient temperature and dark conditions through electron paramagnetic resonance (EPR, JES-FA 200, Japan). The concentration of lithium ions released from the materials was determined using inductively coupled plasma optical emission spectroscopy (ICP-OES, Agilent 5100). Electrochemical measurements were performed using a CHI660E electrochemical analyzer. Approximately 50 mg of the sample was placed in a mortar, followed by the addition of 20 µL of 5% PVDF solution and 20 µL of NMP. The mixture was thoroughly blended and ground into a slurry. This slurry was then uniformly coated onto FTO conductive glass with an area of 1 cm^2^. The coated sample was subsequently dried in an oven at 60°C. The sample electrode, Hg/HgCl electrode and Pt wire served as the working electrode, reference electrode and counter electrode, respectively. A 0.50 mol/L Na_2_SO_4_ solution was used as the electrolyte in all tests.

### Catalytic performance

Since the ROS-generation ability of piezoelectric acoustic sensitizers is positively correlated with their piezocatalytic performance, the piezocatalytic activity of STO and 1.5LSTO powder samples was evaluated by degrading the dye Rhodamine B under ultrasonic vibration conditions (40 kHz, 120 W). Initially, 0.1 g of each powder sample was mixed with 100 mL of RhB solution (10 mg/L) in a beaker. This mixture was stirred with a magnetic stirrer for 40 min in the dark to achieve dissociation-adsorption equilibrium. The beaker was then placed in an ultrasonic bath for piezoelectric catalytic degradation. The entire process was conducted in the dark at ∼25°C, with water circulation employed to minimize interference from light and heat. To assess the piezoelectric catalytic activity, 3 mL of the reaction solution was periodically withdrawn, and the powder was separated by centrifugation. The concentration of the dye was then determined by measuring the absorbance of RhB at 554 nm using a UV–Visible spectrophotometer.

In addition, the piezoelectric catalytic rate constant of the sample was calculated based on the dye degradation results to further illustrate the piezoelectric catalytic ability. The catalytic reaction generally adheres to the Langmuir–Hinshelwood model [28], as illustrated by equation presented below:


(1) $$C=C_{0}\times e^{-kt}$$

In this Equation (1), *t* and *k* denote the reaction time and the pseudo-first-order rate constant, respectively.

### Cellular toxicity

Mouse colorectal carcinoma cells (CT26) and mouse fibroblasts (3T3) were seeded in dishes at a density of 1 × 10^4^ cells per dish in DMEM medium supplemented with 10% fetal bovine serum and 1% penicillin-streptomycin, and cultured in an incubator at 37°C with 5% CO_2_ for 24 h to allow cell attachment. To evaluate the cytotoxicity of the materials, CT26 and 3T3 cells were detached using 0.25% trypsin and seeded at a density of 1 × 10^4^ cells per well in 96-well plates, followed by a 24 h incubation for cell attachment. Subsequently, the cells were co-incubated with graded concentrations (0, 5, 10, 20, 40, 80, 120, 160 mg/L, *n* = 5) of STO/1.5LSTO for 24 h. After incubation, the medium was aspirated, and the wells were gently rinsed three times with PBS. Freshly prepared MTT solution was then added, and the cells were incubated for an additional 4 h. The liquid in the wells was discarded, and dimethyl sulfoxide (DMSO) was added, followed by shaking for 10 min. Absorbance at 490 nm was measured using a microplate reader to assess cell viability, which was compared to the control group (no material). Additionally, live-dead dye was added to the cells treated with the samples, and cell staining was observed using an inverted fluorescence microscope.

### Piezocatalytic therapy of tumor cells *in vitro*

CT26 cells were seeded at a density of 1 × 10^4^ cells per well in 6-well plates and incubated for 24 h to allow cell attachment. Groups designated for sonication were incubated with cells in cell culture dishes. Following this, STO and 1.5LSTO were added to the wells. After a total incubation period of 8 h, ultrasound irradiation (1.0 MHz, 1.0 W/cm^2^, 5 min) was applied to the groups designated for sonication. The cells were then stained with live-dead dyes to assess cell viability. Additionally, the cells were treated with MTT, and the absorbance at 490 nm was measured using a microplate reader to further evaluate the *in vitro* treatment effects. To measure ROS production, the procedure was similar to the Calcein-AM/PI staining method described above, except that the cells were pre-treated with DCFH-DA (1000 μL, 10% in the culture medium). Subsequently, CT26 cells were irradiated with ultrasound (1.0 MHz, 1.0 W·cm^−2^, 5 min) and incubated for an additional 1 h. Finally, the cells were washed three times with PBS and observed using a confocal laser scanning microscope (CLSM).

### Animal experiment

Female BALB/c mice (5–7 weeks) were used in the following animal experiments. All animal studies complied with the animal protocols approved by the Institutional Animal Care and Use Committee (IACUC Approval number: SCU42-2407-02) of the Animal Experiment Center of Sichuan University (Chengdu, China). The tumor models were established by subcutaneously injecting CT26 cells (1 × 10^6^ per mouse, dispersed in 0.1 mL PBS) into the BALB/c mice. Tumor volume and body weight were recorded every two days. Tumor volume (*V*) was calculated using the formula [*V* = (*ab*^2^)/2, where a and b represent the maximum length and width of the tumor, respectively]. Once tumor volumes reached ∼100 mm^3^, the tumor-bearing mice were randomly divided into five groups (*n* = 4): (i) Control group, (ii) STO group, (iii) 1.5LSTO group, (iv) STO + US group and (v) 1.5LSTO + US group. Each group of mice received an intravenous injection of 100 μL of 80 mg/L STO or 1.5 LSTO solution. For the ultrasound-treated groups, tumors were irradiated with ultrasound (1.0 MHz, 1.0 W/cm^2^, 50% duty cycle, 5 min) on Days 1, 3, 5 and 7 postinjection. At the end of the experiment, blood samples, solid tumors and major organs (heart, liver, spleen, lungs and kidneys) were collected for histopathological analysis and blood biochemical assays.

## Result and discussion

### Structural and compositional characterization

**Figure 1:**
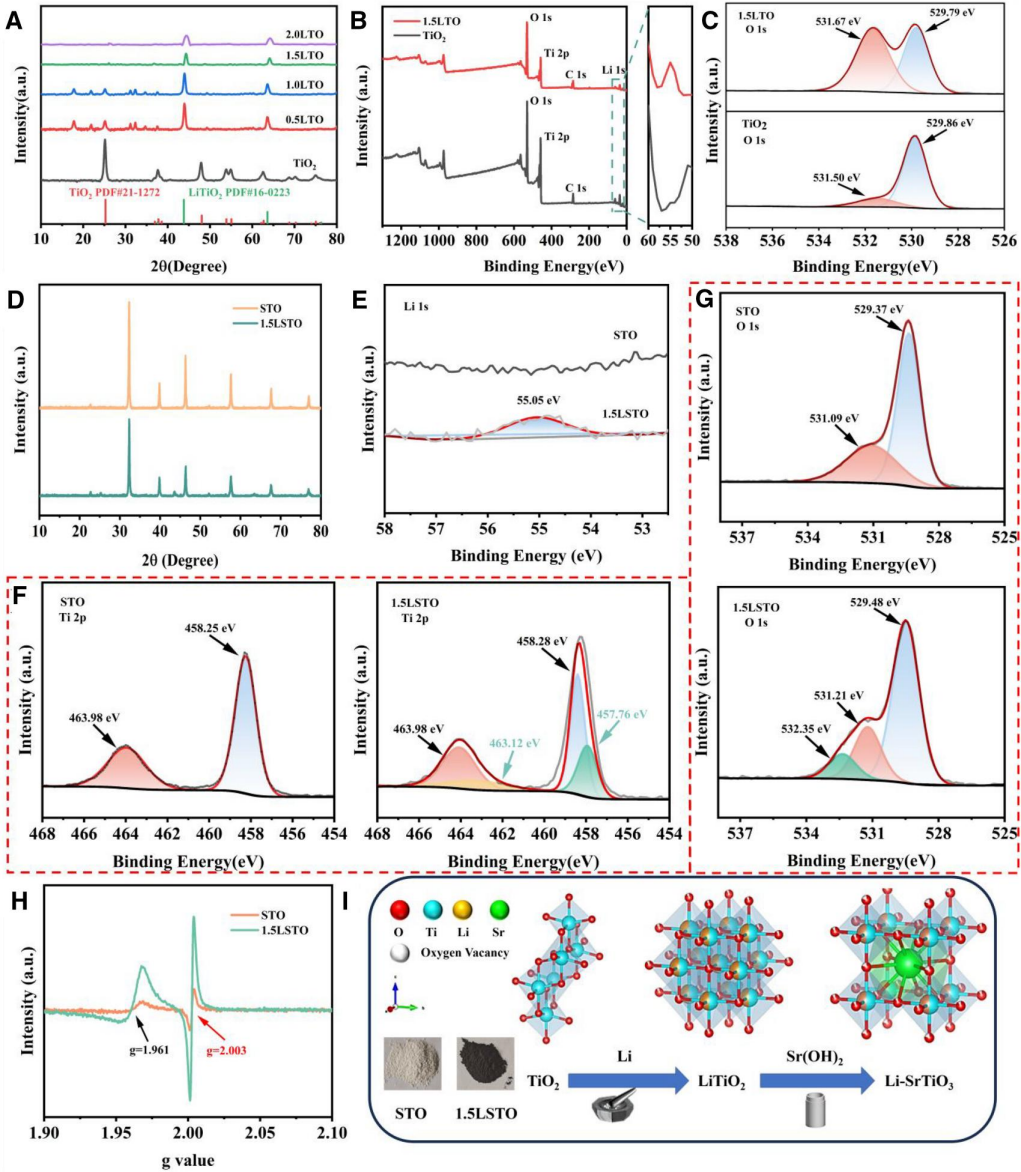
(**A**) XRD spectra of TiO_2_, 0.5LTO, 1.0LTO, 1.5LTO and 2.0LTO powders. (**B**) XPS full spectra of 1.5LTO and TiO_2_. (**C**) O 1s fitting results for split peaks of 1.5LTO and TiO_2_. (**D**) XRD spectra of STO and 1.5LSTO powders. (**E**) Li 1s fitting results for split peaks of STO and 1.5LSTO. (**F**) Ti 2p fitting results for split peaks of STO and 1.5LSTO. (**G**) O 1s fitting results for split peaks of STO and 1.5LSTO. (**H**) EPR spectra of STO and 1.5LSTO powders. (**I**) Schematic diagram of the changes in crystal structure during synthesis and the digital photographs of the samples.

The phase evolution of Li-doped TiO_2_, synthesized via solid-state grinding, was first investigated. A visible darkening of the nanoparticles was observed with increasing Li doping concentration (Supplementary Figure S1), which might be attributed to the enhanced visible-light absorption resulting from the reduction of TiO_2_ by metallic lithium, a potent reducing agent that reacted with lattice oxygen [29, 30]. To elucidate the underlying structural changes, XRD analysis was performed on the pristine and Li-reduced TiO_2_ samples (denoted as 0.5LTO, 1.0LTO, 1.5LTO and 2.0LTO). As shown in Figure 1A, the pristine TiO_2_ exhibited a pure anatase phase. Upon lithium reduction, distinct lithium titanate phases emerged alongside the residual TiO_2_. Notably, the concentration of the rock salt-type LiTiO_2_ phase increased progressively with the Li: Ti molar ratio. This phase became dominant at a ratio of 1:1 and eventually replaced the anatase phase almost completely at a ratio of 1.5:1. This phase transformation is likely driven by the solid-state diffusion of Li^+^ ions into the TiO_2_ lattice during grinding. The intercalation of lithium ions subsequently induces a structural reorganization, culminating in the phase transition. The overall reaction can be represented as follows:


(2) $${\mathrm{TiO}}_{2}\;+\;{\mathrm{xLi}}^{+}+{\mathrm{xe}}^{-}={{\mathrm{Li}}_{x}T\mathrm{iO}}_{2}$$

The parameter *x* in Equation (2) represented the Li-to-TiO_2_ molar ratio. In line with the literatures [31, 32], the value of *x* was varied from 0.5 to 1. Throughout this lithiation process, Li^+^ ions and Ti^3+^ species coexisted within the TiO_2_ crystal structure. When the Li: Ti ratio was further increased to 2:1, no new crystalline phases emerged; however, the LiTiO_2_ diffraction peaks exhibited a decrease in intensity alongside a concomitant increase in the half-peak width. This phenomenon suggests that excess lithium metal participated in the reaction, which could disrupt the long-range structural order and introduce a significant density of crystal defects. Consequently, a Li: Ti ratio of 1.5 appears to represent an optimal threshold for lithium–ion intercalation without inducing excessive structural degradation.

To further verify the existence of surface oxygen vacancies, the surface chemical states of the pristine TiO_2_ and 1.5LTO nanoparticles were examined by XPS. The survey spectra (Figure 1B and Supplementary Figure S2) confirmed the presence of lithium in the 1.5LTO sample, providing direct evidence for the successful incorporation of Li^+^ ions into the crystal lattice to form LiTiO_2_ during the grinding process.

Furthermore, the high-resolution O 1 s spectra at 529.80 and 531.67 eV corresponds to different states of lattice oxygen and oxygen vacancies, respectively (Figure 1C). The pronounced intensity of the oxygen vacancy peak in 1.5LTO unequivocally indicates that the reduction by metallic lithium effectively removes lattice oxygen, generating numerous oxygen vacancies. These vacancies are known to increase the charge carrier concentration, thereby enhancing its catalytic performance [33, 34]. Based on these findings, which demonstrate an optimal level of lithium incorporation and defect generation, the 1.5LTO sample was selected as the precursor for the subsequent solid-state grinding synthesis of LSTO.

To evaluate the impact of lithium doping on the piezocatalytic performance of strontium titanate (STO), both undoped STO and Li-doped STO (1.5LSTO) samples were synthesized. The XRD patterns (Figure 1D) reveal that all powder samples are highly crystalline. Notably, the diffraction peak intensities of 1.5LSTO are lower than those of pure STO. This reduction in intensity can be ascribed to lattice distortions induced by the incorporation of Li^+^ into the TiO_2_ crystal structure during synthesis [35, 36]. Specifically, the ionic radius of Li^+^ (0.76 Å) is significantly larger than that of Ti^4+^ (0.61 Å). Substitution of Ti^4+^ by Li^+^, thus, introduces local lattice strain, modifying the crystal structure factor. Furthermore, the intercalation and deintercalation of Li^+^ ions can lead to variations in lattice parameters [37, 38]. Additionally, lithium doping introduces oxygen vacancies into the lattice during the reduction process, which further influences the overall crystallinity. Collectively, these structural modifications directly diminish the intensity of the XRD diffraction peaks. To confirm the chemical state of the Li dopant and assess its impact on the electronic structure, the Li 1s binding energy peaks of the samples before and after doping were subjected to peak deconvolution and fitting (Figure 1E). The fitting results show that there was a pronounced Li 1s feature in the 1.5LSTO sample, directly demonstrating that lithium has been successfully incorporated into the SrTiO_3_ lattice.

To further elucidate the impact of lithium doping on the crystal structure, XPS was employed to examine changes in the chemical states of Ti and O before and after doping. The Ti 2p binding energy peaks of undoped and Li-doped SrTiO_3_ were subjected to segmented fitting (Figure 1F). For pristine STO, a pair of splitting peaks was observed, with the Ti 2p_3/2_ peak located at 458.25 eV and the Ti 2p_1/2_ peak at 463.96 eV, characteristic of Ti^4+^ [39]. In contrast, the spectra of 1.5LSTO revealed two distinct doublets: one set corresponding to Ti^4+^ (Ti 2p_3/2_ at 458.28 eV and Ti 2p_1/2_ at 463.98 eV), and another set attributable to Ti^3+^ (Ti 2p_3/2_ at 457.76 eV and Ti 2p_1/2_ at 463.12 eV). Previous studies have demonstrated that the higher content of the Ti^3+^, the higher intensity of the corresponding band-gap intermediate states. These intermediate states effectively narrow the bandgap and act as electron traps, suppressing the recombination of electron–hole pairs. As a result, carrier lifetimes are prolonged, and surface reaction activity is enhanced [40, 41]. Therefore, the increased Ti^3+^ content in 1.5LSTO is expected to contribute to improved catalytic performance. The O 1s spectra at 529.48, 531.21 and 532.35 eV correspond to lattice oxygen, oxygen vacancies and surface adsorbed oxygen, respectively (Figure 1G). A pronounced oxygen vacancy peak was evident in 1.5LSTO, whereas no such feature was detected in undoped STO. This indicates that lithium reduction during synthesis consumed lattice oxygen and generates a substantial number of oxygen vacancies. And the introduction of these vacancies increases the carrier concentration in the material, thereby further enhancing its catalytic activity.

The presence of oxygen vacancies was further confirmed through EPR measurements, as shown in Figure 1H. It has been established that EPR signals within the range of *g* = 1.960–1.990 originated from Ti^3+^ sites [42, 43]. In the case of 1.5LSTO, a characteristic signal was observed at *g* = 1.961, confirming the incorporation of Ti^3+^ during the synthesis process. Moreover, compared with undoped STO, the 1.5LSTO sample exhibited a stronger EPR signal at *g* = 2.003, which is attributable to electrons localized in oxygen vacancies [44, 45]. This result not only confirms the presence of oxygen vacancies but also indicates an increase in their concentration following lithium doping. Based on the comprehensive phase analysis, the structural evolution during material synthesis was elucidated. As illustrated in Figure 1I, a series of characterizations confirmed that the crystallization pathway proceeds from anatase TiO_2_ to rock-salt-type LiTiO_2_, and finally, to the perovskite-structured Li-doped SrTiO_3_.

### Microstructure and lattice characteristics

**Figure 2:**
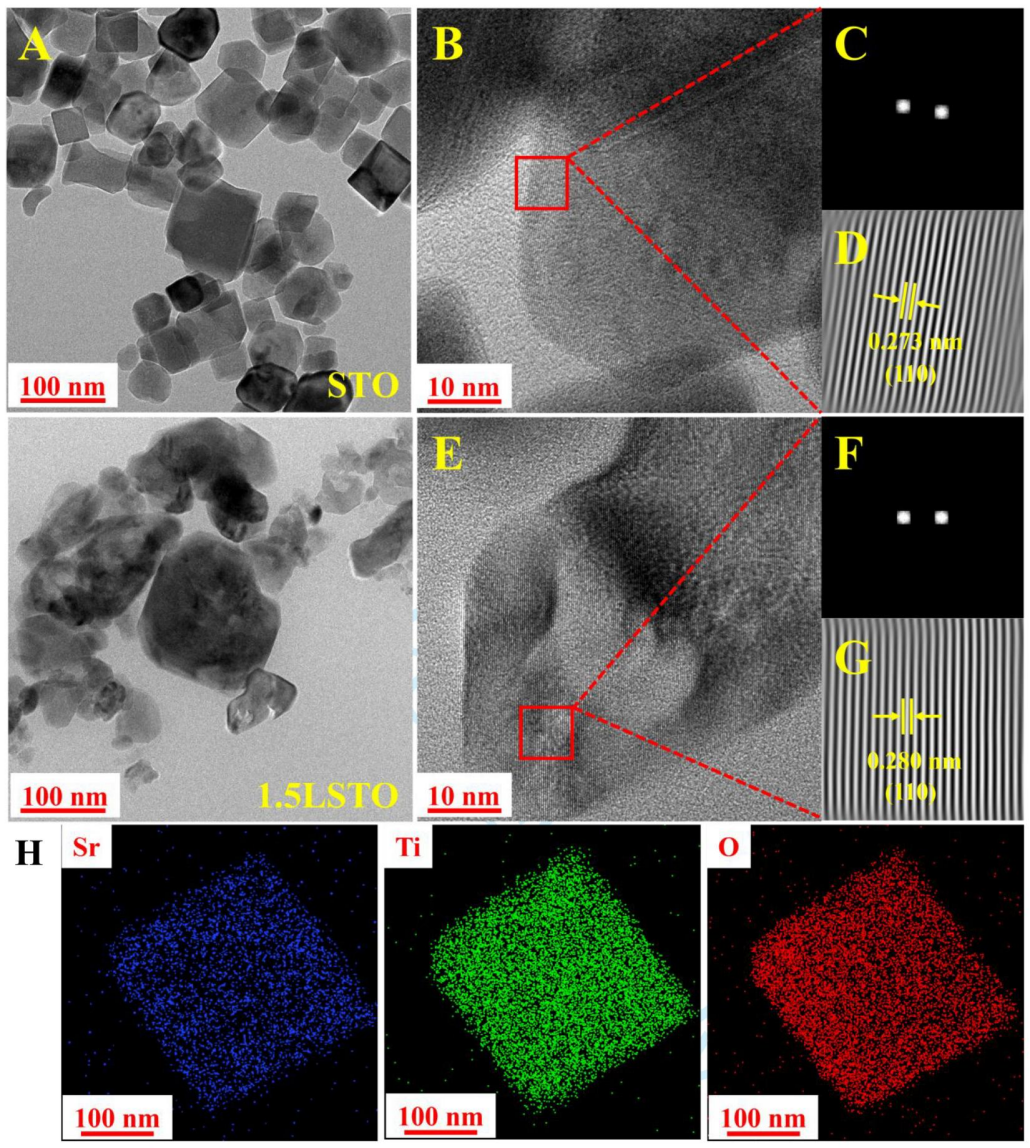
(**A**) TEM images of STO and 1.5LSTO. (**B**) HRTEM images of STO. (**C**) FFT and (**D**) IFFT images of the selected region in the STO. (**E**) HRTEM images of 1.5LSTO. (**F**) FFT and (**G**) IFFT images of the selected region in the STO. (**H**) EDX-STEM mapping of 1.5LSTO.

TEM was employed to characterize the microstructural features of the samples systematically. As shown in Figure 2A, the particle size ranges of both STO and 1.5LSTO samples were ∼20–80 nm. In terms of morphology, undoped STO particles predominantly exhibited a regular cubic shape, whereas 1.5LSTO particles display a more irregular morphology, indicating that lithium doping induced significant morphological changes. This transformation can be attributed to the solid-state grinding method used to prepare the Li-doped precursor, which modified the surface energy of the crystallites during the formation of SrTiO_3_. Concurrently, the introduction of oxygen vacancies led to the formation of edge defects, further influencing the crystal growth trajectory [46, 47].

HRTEM, complemented by fast Fourier transform (FFT) and inverse FFT (IFFT) analyses, was performed to elucidate the crystalline structures of STO and 1.5LSTO nanocrystals. FFT (Figure 2C and F) and IFFT (Figure 2D and G) patterns were obtained from selected regions in Figure 2B and E, respectively, to clarify the atomic-scale structural features. Analysis confirmed that the STO nanocrystal was single-crystalline, exhibiting well-defined lattice fringes with a measured interplanar spacing of 0.273 nm, corresponding to the (110) plane (Figure 2D). In contrast, the 1.5LSTO sample showed a lattice spacing of 0.280 nm along the same orientation (Figure 2G), indicating a slight but discernible lattice expansion relative to undoped STO. Both samples exhibited isotropic lattice planes (Supplementary Figure S3). The observed lattice expansion can be attributed to the combined effects of Li^+^ incorporation and oxygen vacancy formation. It has been reported that both Li^+^ doping and oxygen vacancies can promote a local structural transition toward a tetragonal phase characterized by *c* > *a* [48, 49]. More importantly, oxygen vacancies induce significant local lattice distortion and swelling in their immediate vicinity. The superposition of vacancy-induced local expansion and elongation along the *c*-axis ultimately leads to an increase in interplanar spacing along specific crystallographic directions [50, 51].

Separately, the SEM was used to observe the surface morphologies of STO and 1.5LSTO. As depicted in Supplementary Figure S4, no significant morphological differences were observed between the undoped and Li-doped samples, indicating that the incorporation of Li^+^ did not substantially alter the external particle surface morphology [52]. The elemental mapping of 1.5LSTO (Figure 2H) further confirmed the homogeneous distribution of Sr, Ti and O throughout the microstructure. However, due to the low X-ray energy of Li, which falls below the detection limit of the EDS system, the spatial distribution of lithium could not be directly resolved. To verify the successful incorporation of Li into the crystal lattice, ICP-OES was subsequently employed to quantify the concentration of lithium ions released from the material (Supplementary Table S1). These quantitative results, combined with the Li 1s peak-fitting data of the 1.5LSTO nanoparticles, can jointly confirm that Li has been successfully incorporated into the sample lattice.

### Piezoelectric property and mechanism

**Figure 3:**
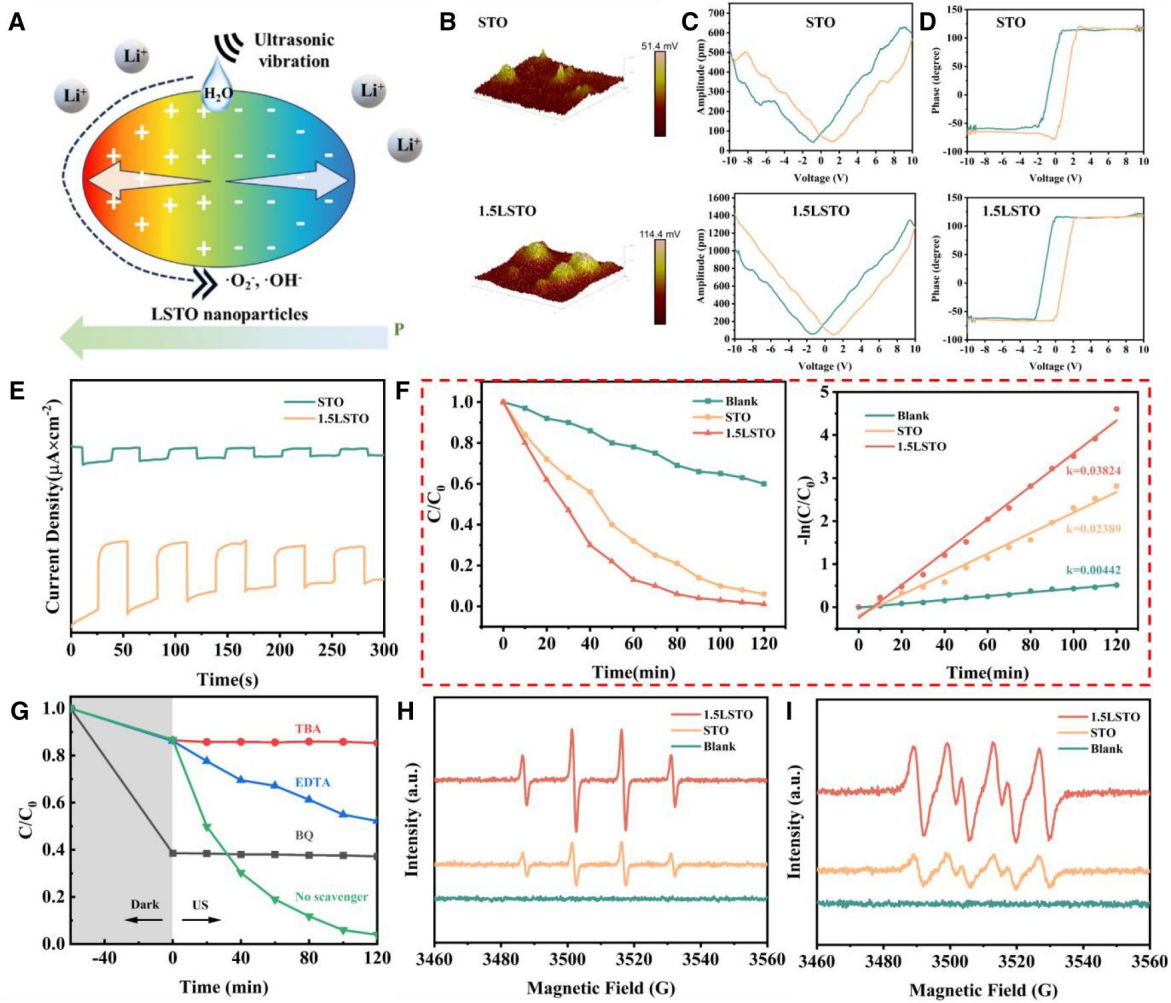
(**A**) Schematic diagram of the ROS generation mechanism. (**B**) Surface-potential maps of STO and 1.5LSTO nanoparticles. (**C**) Amplitude–voltage and (**D**) phase-voltage diagrams of materials, confirming their piezoelectricity. (**E**) Transient piezoelectric response currents. (**F**) Piezocatalytic degradation efficiency and kinetic rate constants for the piezocatalytic degradation reactions of STO and 1.5LSTO powder samples. (**G**) Piezocatalytic degradation efficiency; DMPO spin-trapping ESR detection of (**H**) DMPO-•OH and (**I**) DMPO-•$O_{2}^{\;-}$.

In general, the catalytic activity of piezoelectric materials is fundamentally governed by the efficient separation and migration of electrons and holes triggered by the piezoelectric effect [53, 54]. As schematically illustrated in Figure 3A, Li-doped SrTiO_3_ nanoparticles undergo polarization under US, leading to redox reactions with water that yield ROS (e.g. •OH and •$O_{2}^{\;-}$) alongside the release of lithium ions. The concentration of released Li^+^ was quantitatively measured, with 1.5LSTO exhibiting a value of ∼10.02 mg/L (Supplementary Table S1). To experimentally validate the proposed mechanism, a series of performance evaluations were subsequently conducted.

The surface potential and piezoelectric properties of the nanoparticles were subsequently characterized by PFM. As shown in Figure 3B, the surface potential increases from 51.4 mV for undoped STO to 114.4 mV for 1.5LSTO. corresponding to an ∼2.23-fold enhancement, which indicates improved piezoelectric performance due to Li doping. Figure 3C and D present the amplitude–voltage butterfly loops and piezoresponse hysteresis curves, respectively, which reveal distinct switching behavior under applied voltage. Both samples exhibit pronounced butterfly-shaped amplitude–voltage curves and characteristic phase–voltage hysteresis loops, characteristic of ferro/piezoelectric materials. Notably, the piezoresponse amplitude of 1.5LSTO (1414.7 pm) is significantly larger than that of undoped STO (597.6 pm). These results confirm that Li doping markedly enhances the piezoelectric properties of SrTiO_3_. This enhancement likely arises from modifications in the electronic structure that facilitate carrier mobility and charge transport, thereby increasing the piezoresponse under mechanical stimulation.

Next, the transient response of the material to piezoelectric current were examined. As shown in Figure 3E, the piezoelectric current density of the undoped STO samples was relatively low, whereas that of 1.5LSTO was significantly enhanced. And this enhancement can be attributed to the increased structural asymmetry induced by Li^+^ doping, which enhances the piezoelectric polarization of the material [55, 56]. Besides, the introduced oxygen vacancies serve as hole traps, suppressing the recombination of charge carriers and thereby promoting more efficient electron–hole separation. This synergistic effect further contributes to the observed increase in piezoelectric current density.

The piezocatalytic activities of the as-prepared powder samples were evaluated by examining the degradation of RhB under US (Figure 3F). Here, C_0_ and C denote the initial and real-time concentrations of RhB, respectively [57]. All experiments were conducted in the dark at room temperature to minimize the interference from thermal and photolytic effects. After 120 min of ultrasonication, both samples demonstrated effective RhB degradation. The degradation efficiency increased progressively with the introduction of lithium doping and associated oxygen vacancies. Notably, 1.5LSTO exhibited superior catalytic performance, achieving over 90% RhB degradation within 80 min, whereas undoped STO required 120 min to reach a comparable level.

In this Equation (1), the relationship between -ln(C/C_0_) and reaction time (*t*) was subjected to linear regression analysis to determine the corresponding apparent rate constant (*k*), as depicted in Figure 3F. The calculated rate constant for RhB degradation for STO was 0.02389 min^−1^, while that for 1.5LSTO reached ∼0.03824 min^−1^. These results indicate that the piezocatalytic degradation rate was enhanced by a factor of ∼1.6 after lithium doping.

Under US, the cavitation effect in the liquid medium facilitates the activation of piezoelectric catalysts, enabling them to react with dissolved oxygen and water molecules to generate •$O_{2}^{\;-}$ and •OH radicals, which subsequently participate in the degradation of dye molecules [58, 59]. To elucidate the roles of different radicals, trapping experiments with various radical scavengers were conducted. BQ, TBA and EDTA were used as scavengers for •$O_{2}^{\;-}$, •OH and holes (h^+^), respectively [60]. As shown in Figure 3G, the addition of BQ and TBA markedly suppressed the piezocatalytic degradation of RhB, with BQ inducing the most significant inhibition. In contrast, the presence of EDTA only slightly reduced the degradation rate, suggesting that holes are not the dominant active species in this process. The free radical trapping efficiencies at varying scavenger concentrations are further summarized in Supplementary Figure S5. These results demonstrate that •$O_{2}^{\;-}$ and •OH serve as the primary reactive species, with •$O_{2}^{\;-}$ playing a more decisive role than •OH.

To further identify the radical species generated during the piezocatalytic process, EPR spectroscopy combined with spin trapping was employed (Figure 3H and I). In the DMPO-•$O_{2}^{\;-}$ ESR spectrum, four strong signals were observed for 1.5LSTO, confirming the generation of •$O_{2}^{\;-}$ active species during the degradation process, and the peak intensity was markedly higher than that of the STO group. Similarly, the DMPO-•OH ESR spectrum indicated the presence of •OH radicals, with the signal intensity of 1.5LSTO being again substantially greater than that of STO. In both sets of spectra, the ultrasound-only control (Blank) showed no appreciable signal, indicating that the detected ROS originated predominantly from the stimulation of the piezoelectric material by ultrasound rather than from ultrasound itself.

These results demonstrate that ultrasonic excitation induces a piezoresponsive built-in electric field within the material, which drives the continuous separation and migration of electrons and holes. The resulting band bending promotes the reduction of oxygen by electrons to form •$O_{2}^{\;-}$ and the oxidation of water by holes to yield •OH, thereby facilitating the degradation process.

### *In vitro* piezoelectric-catalyzed antitumor effects

**Figure 4:**
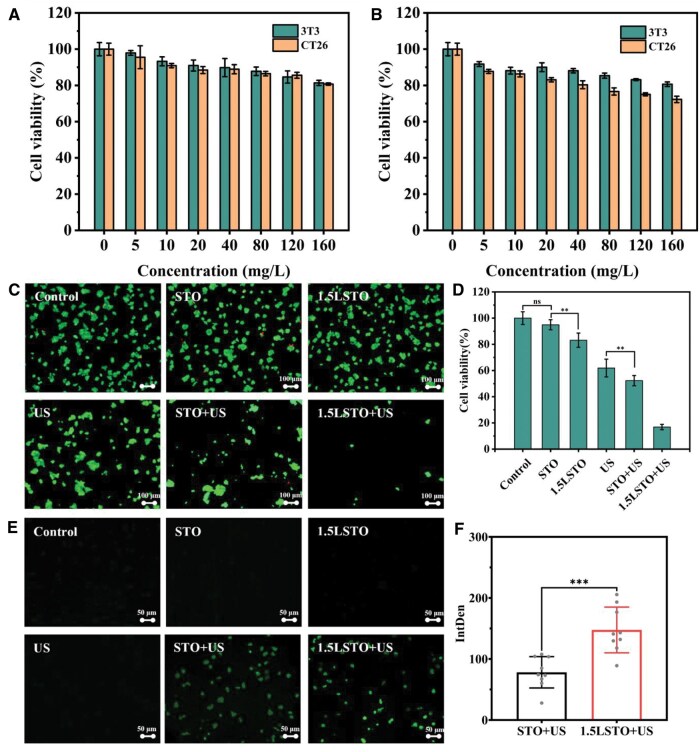
(**A**) Cell viability of 3T3 and CT26 cells after a 24-hour incubation with STO at varying concentrations (0–160 mg/L). (**B**) Cell viability of 3T3 and CT26 cells after a 24-h incubation with 1.5LSTO at varying concentrations (0–160 mg/L). (**C**) Representative fluorescence images of live/dead cell staining for CT26 cells after treatments. (**D**) Cell viability of CT26 cells after treatments. (**E**) Representative fluorescence images of ROS staining in CT26 cells after different treatments. (**F**) significance analysis of ROS fluorescence intensity (*n* = 9, significance levels denoted by ***P < 0.001, **P < 0.01, ns means no significance, one-way ANOVA with Tukey’s *post hoc* test).

The previous section discussed the fundamental principle and potential mechanism underlying ROS generation via piezoelectric catalysis. Subsequently, we further evaluated the catalytic therapeutic efficacy of the prepared SrTiO_3_ piezoelectric catalysis at the cellular level. The cytotoxicity of the materials was firstly evaluated using the standard 3-(4,5-Dimethylthiazol-2-yl)-2,5-diphenyltetrazolium bromide (MTT) assay, where NIH 3T3 cells (a murine fibroblast cell line) and CT26.WT cells (a murine colon cancer cell line) were co-cultured with the synthesized materials for 24 h. Even at concentrations up to 80 mg/L, both STO and 1.5LSTO exhibited negligible cytotoxicity (Figure 4A and B). Notably, at equivalent material concentration, CT26.WT cells showed lower viability compared to NIH 3T3 cells, an effect potentially attributable to the antitumor activity of lithium ions, which may selectively impair cancer cell proliferation, as supported by prior research [61]. To further assess the cytotoxic profile of the material, Calcein-AM (staining live cells green) and propidium iodide (PI, staining dead cells red) were employed for fluorescence-based viability staining. Fluorescence microscopy images (Supplementary Figures S6–S9) showed that significant cytotoxicity emerged at higher nanoparticle concentrations, characterized by increased red fluorescence. In contrast, at 80 mg/L, the fluorescence signals were overwhelmingly green with only minimal red staining, reflecting a robust and viable cellular layer. Based on these findings, this concentration was selected for all subsequent cellular and *in vivo* experiments.

Subsequently, CT26 cells were utilized as a tumor model to assess the influence of the synthesized materials on cancer cell viability. As depicted in Figure 4C, the viability of the cells co-cultured only with the material did not significantly decrease, indicating that the STO exhibited low intrinsic cytotoxicity. By contrast, co-culture with 1.5LSTO led to a noticeable decrease in viability, likely attributable to the release of Li ions from 1.5LSTO. When combined with ultrasound exposure (1.0 MHz), a markedly stronger suppression of cell viability was observed, indicating a synergistic piezocatalytic effect. The selection of 1.0 MHz as the excitation frequency was based on its established suitability for preclinical tumor models. At this frequency, ultrasound achieves an optimal compromise between tissue penetration depth and spatial resolution in small animals. Under typical soft-tissue attenuation conditions, it provides sufficient energy deposition at depths relevant to subcutaneous murine tumors, while remaining within widely adopted safety and efficacy parameters for sonodynamic and therapeutic ultrasound studies [62, 63].

At a material concentration of 80 mg/L, the cell survival rate was 52% in the STO + US group, whereas it dropped markedly to below 17% in the 1.5LSTO + US group, revealing the superior antitumor efficacy of the latter. The cytotoxic effects of the materials on tumor cells aligned well with the results of the piezoelectric-catalyzed dye degradation, thereby supporting the proposition that ROS generated by the piezoelectric materials under US constitute a crucial mechanism underlying the antitumor effect. Moreover, comparative analysis indicated that ultrasound treatment alone exerted only a minimal therapeutic impact on tumor cells. Additional validation was provided by the live/dead staining images of tumor cells shown in Figure 4D, which consistently reinforced the foregoing experimental conclusions.

These results demonstrated that the prepared material itself exhibited minimal cytotoxicity. However, under US, the materials induced a pronounced cytotoxicity in tumor cells. This phenomenon indicated a direct correlation between the material’s antitumor efficacy and its piezoelectric catalytic activity under US.

Further analysis revealed that US-triggered ROS generation enhanced the piezoelectric catalytic performance of the material, leading to increased tumor cell death (Figure 4E). These findings provide experimental evidence for the critical role of ROS in mediating the antitumor effects of piezoelectric materials. Additionally, quantitative analysis of fluorescence intensity showed a statistically significant difference between the 1.5LSTO + US and STO + US groups (Figure 4F). To sum up, this study indicated that activating piezoelectric materials via US to generate ROS represents a key strategy for effective tumor treatment.

### *In vivo* piezoelectric antitumor effect

**Figure 5:**
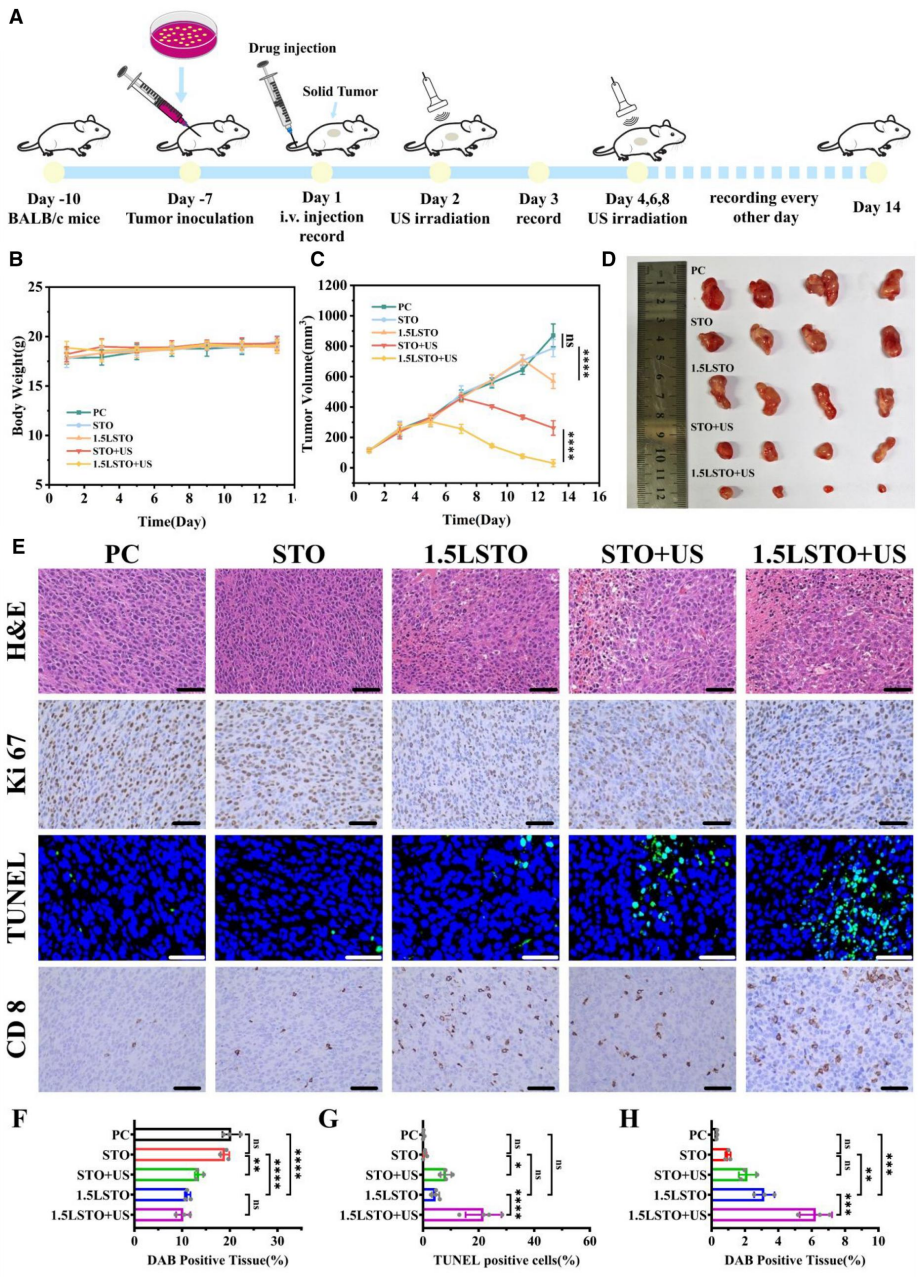
(**A**) Schematic illustration of the treatment timeline for *in vivo* synergistic antitumor therapy. (**B**) Average tumor growth curves of the mice following various therapeutic regimens. (**C**) Body weight trajectories in CT26 tumor-bearing murine models over a 14 days therapeutic regimen. (**D**) Photographs of tumor morphology from different groups after 14 days of therapy. (**E**) H&E, Ki-67, TUNEL and CD8 staining assay of tumor slices on Day 14 (Scale bar = 50 μm); representative immunofluorescence images showing expression levels of (**F**) Ki-67 (proliferation marker), (**G**)TUNEL (apoptosis marker) and (**H**) CD8 (cytotoxic T cell marker). (*n* = 3, significance levels denoted by *P < 0.05, **P < 0.01, ***P < 0.001 ****P < 0.0001, ns means no significance, one-way ANOVA with Tukey’s *post hoc* test).

To evaluate the *in vivo* piezocatalytic antitumor efficacy of the materials, a tumor-bearing mouse model was established through subcutaneous inoculation of CT26 tumor cells in BALB/c mice (Figure 5A). On the first day, all experimental animal groups were administered nanoparticles *via* tail vein intravenous injection. For ultrasound-treated groups, the tumor regions were subjected to US irradiation at Days 1, 3, 5 and 7 postinjection to activate piezocatalytic effects. Notably, no significant body weight fluctuations were observed in any treatment groups throughout the experimental period, preliminarily confirming the biosafety of this therapeutic strategy (Figure 5B). To assess the *in vivo* piezocatalytic antitumor efficacy of the materials, the therapeutic outcome was evaluated based on tumor volume measurements. Among all groups, the 1.5LSTO + US group exhibited the most pronounced suppression of tumor growth, as reflected by the smallest final tumor volume. Moderate antitumor effects were observed in both the STO + US and 1.5LSTO groups, where tumor growth was partially retarded. In contrast, tumors in the PC and STO groups continued to proliferate rapidly, showing no signs of growth inhibition (Figure 5C). Macroscopic evaluation revealed that the 1.5LSTO + US group had the smallest residual tumor volume, corroborating its superior antitumor performance (Figure 5D).

At the experimental endpoint (Day 14), all mice were euthanized, and tumors were harvested for analysis. Histopathological examination by H&E staining revealed marked differences in the extent of tumor necrosis across the experimental groups (Figure 5E). Notably, the 1.5LSTO + US group exhibited the most extensive necrotic areas, accounting for ∼20% of the total tumor cross-sectional area, significantly exceeding that observed in other groups. Morphologically, the necrotic regions were characterized by cytoplasmic dissolution, nuclear pyknosis, karyorrhexis and scattered cellular debris, indicative of irreversible cell injury. These findings demonstrate that the combination of 1.5LSTO with US irradiation synergistically enhances tumoricidal efficacy compared to the PC and 1.5LSTO-alone groups.

Immunohistochemical staining analysis further revealed that Ki-67, a marker of proliferative activity, was most highly expressed in the PC group. In contrast, all treatment groups showed significant suppression of Ki-67 expression, with the lowest level observed in the 1.5LSTO + US group, corroborating its potent antitumor effect (Figure 5E and F). Interestingly, the 1.5LSTO group without ultrasound also exhibited considerable tumor suppression relative to both STO and STO + US groups, suggesting that lithium ions may exert intrinsic antitumor activity through US-independent mechanisms.

TUNEL staining confirmed differential apoptotic responses among the groups, with the highest rate of tumor cell apoptosis occurring in the 1.5LSTO + US group, ∼3.81-fold greater than in the 1.5LSTO-alone group and significantly higher than in the STO + US group. Moreover, lithium doping alone conferred intrinsic pro-apoptotic activity, as evidenced by the enhanced apoptosis in the 1.5LSTO group compared to undoped STO (Figure 5E and G).

Immunohistochemical analysis for CD8^+^ T cells revealed distinct brown–yellow staining within tumor tissue sections. Notably, lithium-doped materials significantly promoted the infiltration of CD8^+^ T cells into the tumor microenvironment. This effect was further enhanced under US, which is consistent with previous studies [64, 65]. As a key metric for assessing antitumor immune responses, the extent of CD8^+^ T cell infiltration confirmed that the combination of lithium doping and US robustly augmented the recruitment of CD8^+^ T lymphocytes to tumor sites (Figure 5E and H). Collectively, these findings indicate a positive correlation between the degree of CD8^+^ T cell infiltration and both tumor necrosis area and apoptosis rate, whereas an inverse correlation was observed with Ki-67 expression, reflecting suppressed tumor proliferation.

The experimental results collectively demonstrated that the piezocatalytic performance of 1.5LSTO was significantly enhanced compared to undoped STO, leading to a substantial increase in ROS generation under US. This optimized ROS production capacity further contributed to the improved antitumor efficacy of the material. Notably, Lithium-doped materials inhibit tumors via three interconnected mechanisms: first, lithium doping optimizes the piezoelectric properties of the material, thereby enhancing ultrasound-driven ROS generation; second, released Li^+^ ions exhibit selective cytotoxicity toward tumor cells while sparing normal cells; and third, Li^+^ promotes the infiltration of CD8^+^ T cells into the tumor microenvironment. Together, these effects establish a synergistic piezocatalytic- immunomodulatory therapeutic strategy. In summary, the 1.5LSTO + US treatment effectively suppressed tumor cell proliferation, promoted CD8^+^ T cells infiltration into tumor tissues, and subsequently enhanced tumor cell apoptosis.

**Figure 6:**
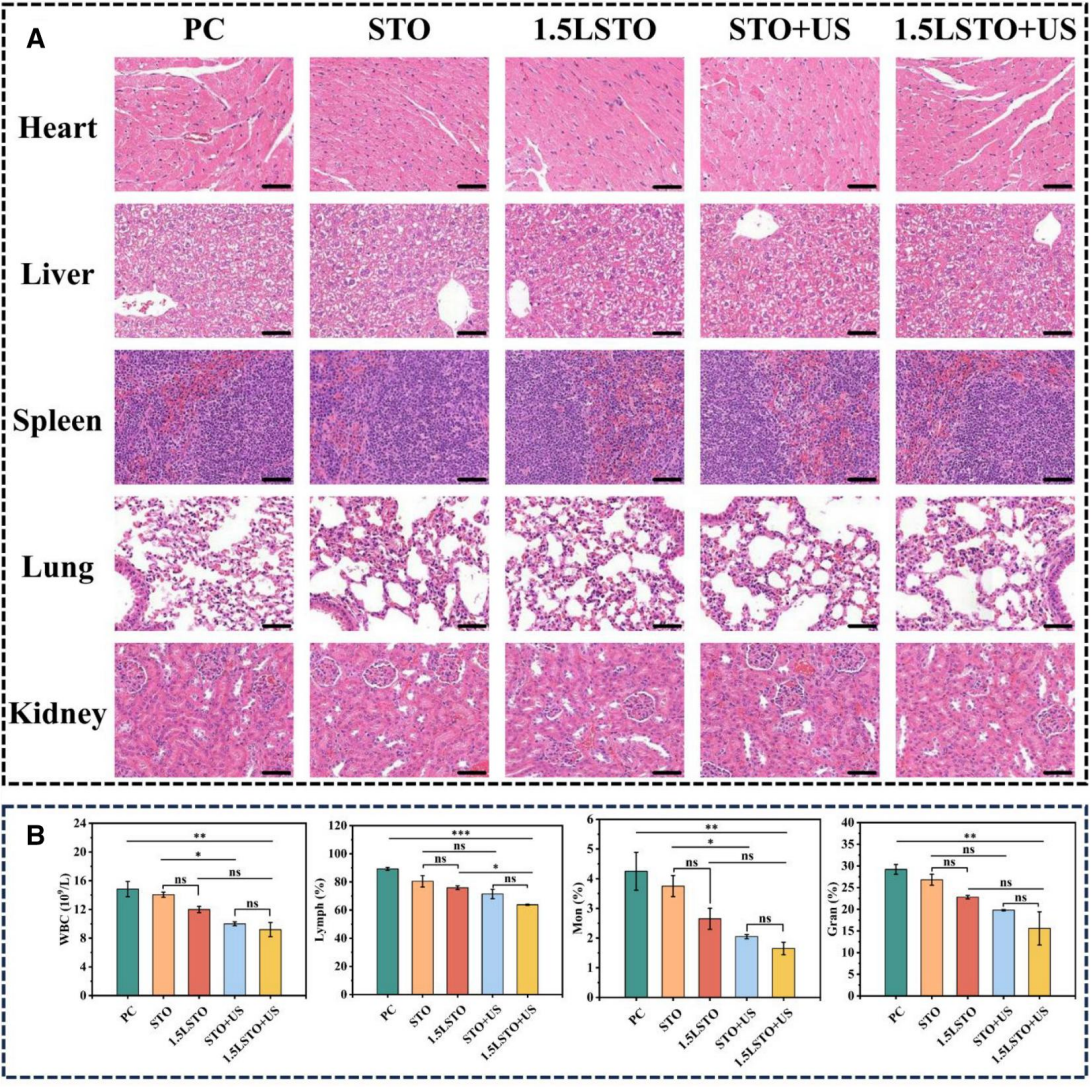
(**A**) Histological sections obtained from the heart, liver, spleen, lung and kidney after various treatments, including PC group, STO group, 1.5LSTO group, STO + US group and 1.5LSTO + US group. (Scale bar = 200 μm); (**B**) Blood biochemistry associated with inflammatory marker on Day 14. (*n* = 3, significance levels denoted by *P < 0.05, **P < 0.01, ***P < 0.001, ns means no significance, one-way ANOVA with Tukey’s *post hoc* test).

Following the complete therapeutic regimen, histopathological analysis of major organs (heart, liver, spleen, lungs and kidneys) was performed by H&E staining, and no apparent tissue damage or inflammatory lesions was found in any experimental groups of PC, STO, 1.5LSTO, STO + US and 1.5LSTO + US (Figure 6A). Additionally, blood samples were collected from tumor-bearing mice after *in vivo* anticancer therapy and analyzed for key biochemical parameters. As illustrated in Figure 6B, compared to other groups, the 1.5LSTO + US group exhibited significantly lower levels of systemic inflammation-related markers—such as white blood cell count (WBC), lymphocyte count (Lymph#), monocyte count (Mon#) and neutrophil count (Gran#). This suppression of inflammatory indices implies that the piezoelectric chemotherapeutic strategy effectively modulates the tumor microenvironment by promoting tumor cell apoptosis. Additional inflammation-related blood biochemistry indicators are shown in Supplementary Figure S10. Furthermore, evaluation of noninflammatory blood markers revealed that all measured values remained within normal reference ranges, as shown in Supplementary Figure S11. Together, these results corroborate the favorable biosafety profile of the proposed treatment *in vivo*.

The experimental findings indicate that the piezocatalytic performance of nanomaterials can be effectively modulated through the element-doping strategy. Notably, lithium incorporation achieves a dual synergistic effect by coupling piezocatalysis with immune activation. This strategy not only enables direct tumor cell eradication via piezoelectric catalysis but also enhances the overall antitumor capacity by harnessing activated immune responses. Consequently, this approach promotes the effective integration of piezocatalytic therapy with tumor immunotherapy.

## Conclusion

In this study, lithium-doped, oxygen-deficient black SrTiO_3_ was successfully synthesized via a low-temperature hydrothermal method using black LiTiO_2_ nanoparticles, integrating elemental doping and defect engineering strategies. Structurally, lithium incorporation induces enhanced internal crystal asymmetry, which facilitates the phase transition of strontium titanate from a centrosymmetric cubic phase to a piezoelectric tetragonal phase. This crystallographic modification substantially augments the piezocatalytic activity, leading to a marked increase in ROS generation under US. Through comprehensive *in vitro* cellular assays and *in vivo* syngeneic tumor models, the engineered nanoparticles demonstrated potent tumor cell eradication under US activation while maintaining excellent biocompatibility in physiological settings. Importantly, lithium doping was shown to confer a dual synergistic effect by coupling piezocatalysis with immune activation. This bifunctional mechanism not only enables direct tumor cell ablation via piezocatalytically generated ROS but also enhances systemic antitumor immunity through promoted CD8^+^ T cell infiltration. The multimodal therapeutic paradigm established herein, based on rational material functionalization, offers a compelling strategy for the development of innovative and synergistic tumor treatment platforms.

## Supplementary Material

rbag030_Supplementary_Data

## References

[1] Eng C , Yoshino T, Ruíz-García E, Mostafa N, Cann CG, O’Brian B, Benny A, Perez RO, Cremolini C. Colorectal cancer. The Lancet 2024;404:294–310. 10.1016/S0140-6736(24)00360-X 38909621

[2] Kaur R , Bhardwaj A, Gupta S. Cancer treatment therapies: traditional to modern approaches to combat cancers. Mol Biol Rep 2023;50:9663–76. 37828275 10.1007/s11033-023-08809-3

[3] Pan Q , Fan X, Xie L, Wu D, Liu R, Gao W, Luo K, He B, Pu Y. Nano-enabled colorectal cancer therapy. J Control Release 2023;362:548–64. 37683732 10.1016/j.jconrel.2023.09.014

[4] Chen S , Zhu P, Mao L, Wu W, Lin H, Xu D, Lu X, Shi J. Piezocatalytic medicine: an emerging frontier using piezoelectric materials for biomedical applications. Adv Mater 2023;35:2208256. 10.1002/adma.202208256 36634150

[5] Jia P , Li J, Huang H. Piezocatalysts and piezo-photocatalysts: from material design to diverse applications. Adv Funct Mater 2024;34:2407309.

[6] Xiang Z , Xu L, Shan Y, Cui X, Shi B, Xi Y, Ren P, Zheng X, Zhao C, Luo D, Li Z. Tumor microenvironment-responsive self-assembly of barium titanate nanoparticles with enhanced piezoelectric catalysis capabilities for efficient tumor therapy. Bioact Mater 2024;33:251–61. 38059123 10.1016/j.bioactmat.2023.11.004 PMC10696196

[7] Wang P , Tang Q, Zhang L, Xu M, Sun L, Sun S, Zhang J, Wang S, Liang X. Ultrasmall barium titanate nanoparticles for highly efficient hypoxic tumor therapy via ultrasound triggered piezocatalysis and water splitting. ACS Nano 2021;15:11326–40. 34180675 10.1021/acsnano.1c00616

[8] Deng R , Zhou H, Qin Q, Ding L, Song X, Chang M, Chen Y, Zhou Y. Palladium-catalyzed hydrogenation of black barium titanate for multienzyme-piezoelectric synergetic tumor therapy. Adv Mater 2024;36:2307568. 10.1002/adma.202307568 37796929

[9] He D , Wang W, Feng N, Zhang Z, Zhou D, Zhang J, Luo H, Li Y, Chen X, Wu J. Defect-modified nano-BaTiO_3_ as a sonosensitizer for rapid and high-efficiency sonodynamic sterilization. ACS Appl Mater Interfaces 2023;15:15140–51. 36929922 10.1021/acsami.2c23113

[10] Tang Y , Bisoyi HK, Chen X-M, Liu Z, Chen X, Zhang S, Li Q. Pyroptosis-mediated synergistic photodynamic and photothermal immunotherapy enabled by a tumor-membrane-targeted photosensitive dimer. Adv Mater 2023;35:2300232. 10.1002/adma.202300232 36921347

[11] Yang B , Chen Y, Shi J. Nanocatalytic medicine. Adv Mater 2019;31:1901778. 10.1002/adma.201901778 31328844

[12] Pan W-L , Tan Y, Meng W, Huang N-H, Zhao Y-B, Yu Z-Q, Huang Z, Zhang W-H, Sun B, Chen J-X. Microenvironment-driven sequential ferroptosis, photodynamic therapy, and chemotherapy for targeted breast cancer therapy by a cancer-cell-membrane-coated nanoscale metal-organic framework. Biomaterials 2022;283:121449. 35247637 10.1016/j.biomaterials.2022.121449

[13] Xu H , Zhang Y, Zhang H, Zhang Y, Xu Q, Lu J, Feng S, Luo X, Wang S, Zhao Q. Smart polydopamine-based nanoplatforms for biomedical applications: state-of-art and further perspectives. Coord Chem Rev 2023;488:215153.

[14] Grupp DE , Goldman AM. Giant piezoelectric effect in strontium titanate at cryogenic temperatures. Science 1997;276:392–4. 9103192 10.1126/science.276.5311.392

[15] Ling J , Wang K, Wang Z, Huang H, Zhang G. Enhanced piezoelectric-induced catalysis of SrTiO_3_ nanocrystal with well-defined facets under ultrasonic vibration. Ultrason Sonochem 2020;61:104819. 31669844 10.1016/j.ultsonch.2019.104819

[16] Karim ME , Shetty J, Islam RA, Kaiser A, Bakhtiar A, Chowdhury EH. Strontium sulfite: a new pH-Responsive inorganic nanocarrier to deliver therapeutic siRNAs to cancer cells. Pharmaceutics 2019;11:89. 30791612 10.3390/pharmaceutics11020089 PMC6410046

[17] Wu Y , He G, Zhang Y, Liu Y, Li M, Wang X, Li N, Li K, Zheng G, Zheng Y, Yin Q. Unique antitumor property of the Mg-Ca-Sr alloys with addition of Zn. Sci Rep 2016;6:21736. 26907515 10.1038/srep21736 PMC4764862

[18] Jin CC , Liu DM, Zhang LX. An emerging family of piezocatalysts: 2D piezoelectric materials. Small 2023;19:e2303586. 37386814 10.1002/smll.202303586

[19] Yang M-M , Luo Z-D, Mi Z, Zhao J, E SP, Alexe M. Piezoelectric and pyroelectric effects induced by interface polar symmetry. Nature 2020;584:377–81. 32814890 10.1038/s41586-020-2602-4

[20] Liu Y , Li Q, Qiao L, Xu Z, Li F. Achieving giant piezoelectricity and high property uniformity simultaneously in a relaxor ferroelectric crystal through Rare-Earth element doping. Adv Sci 2022;9:2204631. 10.1002/advs.202204631 PMC9762314 36285669

[21] Wu B , Zheng H, Wu Y-Q, Huang Z, Thong H-C, Tao H, Ma J, Zhao C, Xu Z, Liu Y-X, Xing Z, Liang N, Yao F-Z, Wu C-F, Wang K, Han B. Origin of ultrahigh-performance barium titanate-based piezoelectrics: stannum-induced intrinsic and extrinsic contributions. Nat Commun 2024;15:7700. 39227599 10.1038/s41467-024-52031-z PMC11371913

[22] Kong J , Li L, Liu J, Marlton FP, Jørgensen MRV, Pramanick A. A local atomic mechanism for monoclinic-tetragonal phase boundary creation in Li-doped Na_0.5_K_0.5_NbO_3_ ferroelectric solid solution. Inorg Chem 2022;61:4335–49. 35239332 10.1021/acs.inorgchem.1c03501

[23] Kim CG , Lee S, Kim M, Cao VA, Kim SY, Nah J. Synergistic enhancement of filtering efficiency and antibacterial performance of a nanofiber air filter decorated with electropolarized lithium-doped ZnO nanorods. ACS Appl Mater Interfaces 2023;15:20977–86. 37070411 10.1021/acsami.3c00744

[24] Villegas-Vázquez EY , Quintas-Granados LI, Cortés H, González-Del Carmen M, Leyva-Gómez G, Rodríguez-Morales M, Bustamante-Montes LP, Silva-Adaya D, Pérez-Plasencia C, Jacobo-Herrera N, Reyes-Hernández OD, Figueroa-González G. Lithium: a promising anticancer agent. Life 2023;13:537. 36836894 10.3390/life13020537 PMC9966411

[25] Ma J , Tang L, Tan Y, Xiao J, Wei K, Zhang X, Ma Y, Tong S, Chen J, Zhou N, Yang L, Lei Z, Li Y, Lv J, Liu J, Zhang H, Tang K, Zhang Y, Huang B. Lithium carbonate revitalizes tumor-reactive CD8^+^ T cells by shunting lactic acid into mitochondria. Nat Immunol 2024;25:552–61. 38263463 10.1038/s41590-023-01738-0 PMC10907288

[26] Zhu B , Yang C, Hua S, Li K, Shang P, Chen X, Hua Z-C. Lithium enhances ferroptosis sensitivity in melanoma cells and promotes CD8^+^ T cell infiltration and differentiation. Free Radic Biol Med 2025;227:233–45. 39645207 10.1016/j.freeradbiomed.2024.12.012

[27] Fadhlina H , Atiqah A, Zainuddin Z. A review on lithium doped lead-free piezoelectric materials. Mater Today Commun 2022;33:104835.

[28] Liu Q , Zhai D, Xiao Z, Tang C, Sun Q, Bowen CR, Luo H, Zhang D. Piezo-photoelectronic coupling effect of BaTiO_3_@TiO_2_ nanowires for highly concentrated dye degradation. Nano Energy 2022;92:106702.

[29] Yang Y , Yin L-C, Gong Y, Niu P, Wang J-Q, Gu L, Chen X, Liu G, Wang L, Cheng H-M. An unusual strong visible-light absorption band in red anatase TiO_2_ photocatalyst induced by atomic hydrogen-occupied oxygen vacancies. Adv Mater 2018;30:1704479. 10.1002/adma.201704479 29315852

[30] Chandrappa S , Galbao SJ, Sankara Rama Krishnan PS, Koshi NA, Das S, Myakala SN, Lee S-C, Dutta A, Cherevan A, Bhattacharjee S, Murthy DHK. Iridium-doping as a strategy to realize visible-light absorption and p-type behavior in BaTiO_3_. J Phys Chem C 2023;127:12383–93.

[31] Wu J , Liu H-W, Tang A, Zhang W, Sheu H-S, Lee J-F, Liao Y-F, Huang S, Wei M, Wu N-L. Unexpected reversible crystalline/amorphous (de)lithiation transformations enabling fast (dis)charge of high-capacity anatase mesocrystal anode. Nano Energy 2022;102:107715.

[32] Yang H-D , Kang Y-Y, Zhu P-P, Chen Q-W, Yang L, Zhou J-P. Facile hydrothermal preparation, characterization and multifunction of rock salt-type LiTiO_2_. J Alloys Compd 2021;872:159759.

[33] Dong H , Zhou Y, Wang L, Chen L, Zhu M. Oxygen vacancies in piezocatalysis: a critical review. Chem Eng J 2024;487:150480.

[34] Dai J , Fan Z, Long Y, Yue W, Huang F, Jiao Y, Deng Y, Chang Y, Wang D. Remarkably boosting piezocatalytic performance of Sr_0.5_Ba_0.5_Nb_2_O_6_ piezoceramics via size optimization and oxygen vacancy engineering. Adv Funct Mater 2024;34:2408754.

[35] Mo H , Chen Q, Wang D, Guo W, Cheng D, Sha Y, Mokhtar MZ, Jia Z, Jacobs J, Thomas AG, Li L, Liu Z, Curry RJ. Laser processing of Li-doped mesoporous TiO_2_ for ambient-processed mesoscopic perovskite solar cells. J Mater Chem C 2024;12:2025–36.

[36] Hattori N , Manseki K, Hibi Y, Nagaya N, Yoshida N, Sugiura T, Vafaei S. Simultaneous Li-doping and formation of SnO_2_-based composites with TiO_2_: applications for perovskite solar cells. Materials 2024;17:2339. 38793406 10.3390/ma17102339 PMC11123386

[37] Park H , Lee W, Thangavel R, Oh W, Jin B-S, Yoon W-S. Enhancing electrochemical performance by triggering a local structure distortion in lithium vanadium phosphate cathode for Li ion batteries. J Mater Chem A 2022;10:25129–39.

[38] Biasi L , Lieser G, Rana J, Indris S, Dräger C, Glatthaar S, Mönig R, Ehrenberg H, Schumacher G, Binder JR, Geßwein H. Unravelling the mechanism of lithium insertion into and extraction from trirutile-type LiNiFeF_6_ cathode material for Li-ion batteries. CrystEngComm 2015;17:6163–74.

[39] Cao B , Liu H, Zhang X, Zhang P, Zhu Q, Du H, Wang L, Zhang R, Xu B. MOF-derived ZnS nanodots/Ti_3_C_2_T_x_ MXene hybrids boosting superior lithium storage performance. Nanomicro Lett 2021;13:202. 34568995 10.1007/s40820-021-00728-x PMC8473522

[40] Zhou H , Zhang D, Xie H, Liu Y, Meng C, Zhang P, Fan F, Li R, Li C. Modulating oxygen vacancies in lead chromate for photoelectrocatalytic water splitting. Adv Mater 2023;35:2300914. 10.1002/adma.202300914 37038704

[41] Wang P , Li X, Fan S, Chen X, Qin M, Long D, Tadé MO, Liu S. Impact of oxygen vacancy occupancy on piezo-catalytic activity of BaTiO_3_ nanobelt. Appl Catal B Environ 2020;279:119340.

[42] Hejazi S , Mohajernia S, Osuagwu B, Zoppellaro G, Andryskova P, Tomanec O, Kment S, Zbořil R, Schmuki P. On the controlled loading of single platinum atoms as a co-catalyst on TiO_2_ anatase for optimized photocatalytic H_2_ generation. Adv Mater 2020;32:1908505. 10.1002/adma.201908505 32125728

[43] Nong S , Dong W, Yin J, Dong B, Lu Y, Yuan X, Wang X, Bu K, Chen M, Jiang S, Liu L-M, Sui M, Huang F. Well-dispersed ruthenium in mesoporous crystal TiO_2_ as an advanced electrocatalyst for hydrogen evolution reaction. J Am Chem Soc 2018;140:5719–27. 29644854 10.1021/jacs.7b13736

[44] Yang X , Cheng J, Li H, Xu Y, Tu W, Zhou J. Self-supported N-doped hierarchical Co_3_O_4_ electrocatalyst with abundant oxygen vacancies for acidic water oxidation. Chem Eng J 2023;465:142745.

[45] Ye J-J , Li P-H, Zhang H-R, Song Z-Y, Fan T, Zhang W, Tian J, Huang T, Qian Y, Hou Z, Shpigel N, Chen L-F, Dou SX. Manipulating oxygen vacancies to spur ion kinetics in V_2_O_5_ structures for superior aqueous zinc-ion batteries. Adv Funct Mater 2023;33:2305659.

[46] Mutter D , Tao C, Urban DF, Elsässer C. Formation energy profiles of oxygen vacancies at grain boundaries in perovskite-type electroceramics. Adv Eng Mater 2023;25:2201847.

[47] Meng W-W , Yan B-L, Xu Y-J. Scalable synthesis of Ti^3+^ self-doped Li_4_Ti_5_O_12_ microparticles as an improved performance anode material for Li-ion batteries. J Alloys Compd 2019;788:21–9.

[48] Li X , Wang J, Zhang S, Sun L, Zhang W, Dang F, Seifert HJ, Du Y. Intrinsic defects in LiMn_2_O_4_: first-principles calculations. ACS Omega 2021;6:21255–64. 34471730 10.1021/acsomega.1c01162 PMC8388003

[49] Zhao T , Zhang J, Wang K, Xiao Y, Wang Q, Li L, Tseng J, Chen M-C, Ma J-J, Lu Y-R, Hirofumi I, Shao Y-C, Zhao X, Hung S-F, Su Y, Mu X, Hua W. Exploring the mechanism of surface cationic vacancy induces high activity of metastable lattice oxygen in Li- and Mn-rich cathode materials. Angew Chem Int Ed Engl 2025;64:e202419664. 39890590 10.1002/anie.202419664

[50] Ziemke CD , Nguyen HM, Amaya-Roncancio S, Gahl J, Xing Y, Heitmann TW, Wexler C. Formation of lattice vacancies and their effects on lithium-ion transport in LiBO_2_ crystals: comparative ab initio studies. J Mater Chem A 2025;13:3146–62.

[51] Li J , Liu X. First-principles study of oxygen vacancies in LiNbO_3_ -type ferroelectrics. RSC Adv 2024;14:9169–74. 38500610 10.1039/d4ra00833b PMC10946246

[52] Kang Y , Pei Y, He D, Xu H, Ma M, Yan J, Jiang C, Li W, Xiao X. Spatially selective p-type doping for constructing lateral WS2 p-n homojunction via low-energy nitrogen ion implantation. Light Sci Appl 2024;13:127. 38821920 10.1038/s41377-024-01477-3 PMC11143290

[53] Cai L , Du J, Han F, Shi T, Zhang H, Lu Y, Long S, Sun W, Fan J, Peng X. Piezoelectric metal–organic frameworks based sonosensitizer for enhanced nanozyme catalytic and sonodynamic therapies. ACS Nano 2023;17:7901–10. 37052950 10.1021/acsnano.3c01856

[54] Lu S , Zhang S, Li L, Liu C, Li Z, Luo D. Piezoelectric effect-assisted Z-scheme heterojunction ZnIn_2_S_4_/BaTiO_3_ for improved photocatalytic reduction of CO_2_ to CO. Chem Eng J 2024;483:149058.

[55] Yu C , Lan S, Cheng S, Zeng L, Zhu M. Ba substituted SrTiO_3_ induced lattice deformation for enhanced piezocatalytic removal of carbamazepine from waterBa. J Hazard Mater 2022;424:127440. 34879510 10.1016/j.jhazmat.2021.127440

[56] Zhang T , Li Y, Li L, Dong X, Chen J, Mu X, Zhang C, Chen Z, Gong W, Li T, Zhang T, Cong S, Zhao Z. Symmetry-breaking triggered by atomic tungsten for largely enhanced piezoelectric response in hexagonal boron nitride. Nano Energy 2022;99:107375.

[57] Huang T-H , Espino FKC, Tian X-Y, Widakdo J, Austria HFM, Setiawan O, Hung W-S, Pamintuan KRS, Leron RB, Chang C-Y, Caparanga AR, Lee K-R, Lai J-Y. Piezocatalytic property of PVDF/graphene self-assembling piezoelectric membrane for environmental remediation. Chem Eng J 2024;487:150569.

[58] Liu G , Zhao T, Wu J, Chang M, Fei H, Li F, Yang S, Li Q. Enhanced removal and selective conversion for NO with N-vacancies g-C_3_N_4_\BaTiO_3_ by piezo-photocatalysis. Sep Purif Technol 2025;360:130914.

[59] Liu Y , Xu H-Y, Li B, Cao M-C, Jin L-G, Shan L-W, Dong L-M. Mechanical-vibration-driven piezocatalytic degradation of organic pollutants over microcrystalline SnSe with selenium vacancies. Chem Eng J 2024;497:155641.

[60] Tan G , She L, Liu T, Xu C, Ren H, Xia A. Ultrasonic chemical synthesis of hybrid mpg-C_3_N_4_/BiPO_4_ heterostructured photocatalysts with improved visible light photocatalytic activity. Appl Catal B: Environ 2017;207:120–33.

[61] Zanni G , Goto S, Fragopoulou AF, Gaudenzi G, Naidoo V, Di Martino E, Levy G, Dominguez CA, Dethlefsen O, Cedazo-Minguez A, Merino-Serrais P, Stamatakis A, Hermanson O, Blomgren K. Lithium treatment reverses irradiation-induced changes in rodent neural progenitors and rescues cognition. Mol Psychiatry 2021;26:322–40. 31723242 10.1038/s41380-019-0584-0 PMC7815512

[62] Wang Q , Tian Y, Yao M, Fu J, Wang L, Zhu YJAM. Bimetallic organic frameworks of high piezovoltage for sono‐piezo dynamic therapy. Adv Mater 2023;35:2301784. 10.1002/adma.202301784 37432882

[63] Liao Y , Wang D, Zhu S, Zhou R, Rahbarizadeh F, Gu Z. Piezoelectric materials for synergistic piezo- and radio-catalytic tumor therapy. Nano Today 2022;44:101510.

[64] Rix A , Heinrichs H, Porte C, Leenaars C, Bleich A, Kiessling F. Ultrasound-induced immune responses in tumors: a systematic review and meta-analysis. J Control Release 2024;371:146–57. 38777126 10.1016/j.jconrel.2024.05.030

[65] Liu S , Zhang Y, Liu Y, Wang W, Gao S, Yuan W, Sun Z, Liu L, Wang C. Ultrasound-targeted microbubble destruction remodels tumour microenvironment to improve immunotherapeutic effect. Br J Cancer 2023;128:715–25. 36463323 10.1038/s41416-022-02076-y PMC9977958

