# Supplementary material for: Synergistic piezo-immunotherapy enabled by lithium-doped SrTiO3 nanocatalysts for potent tumor ablation through ROS generation and immune activation
Source: Regen Biomater. 2026 Mar 24;13:rbag030. doi: 10.1093/rb/rbag030 (PMC13075956; doi:10.1093/rb/rbag030)
Supplement: rbag030_Supplementary_Data [file rbag030_supplementary_data.docx]

**Synergistic Piezo-Immunotherapy Enabled by Lithium-Doped SrTiO_3_ Nanocatalysts for Potent Tumor Ablation through ROS Generation and Immune Activation**

Yuzheng Gao ^a^, Yichun Zhang^a^, Shuyan Zhang ^a^, Zhiwei Yang^b^, Wenjing Liu ^a^, Jing Zhang^b^, Zhipeng Gu^a^*, Xianchun Chen ^a^*

*^a^* College of Polymer Science and Engineering, Sichuan University, No.24 South Section 1, Yihuan Road, Chengdu, 610065, China

*^b^* College of Architecture and Environment, Sichuan University, Chengdu, 610065, China

**Corresponding Authors**

1. Zhipeng Gu, E-mail: guzhipeng2019@scu.edu.cn
2. Xianchun Chen, E-mail: chenxianchun@scu.edu.cn

**Notes**

The authors declare no competing financial interest


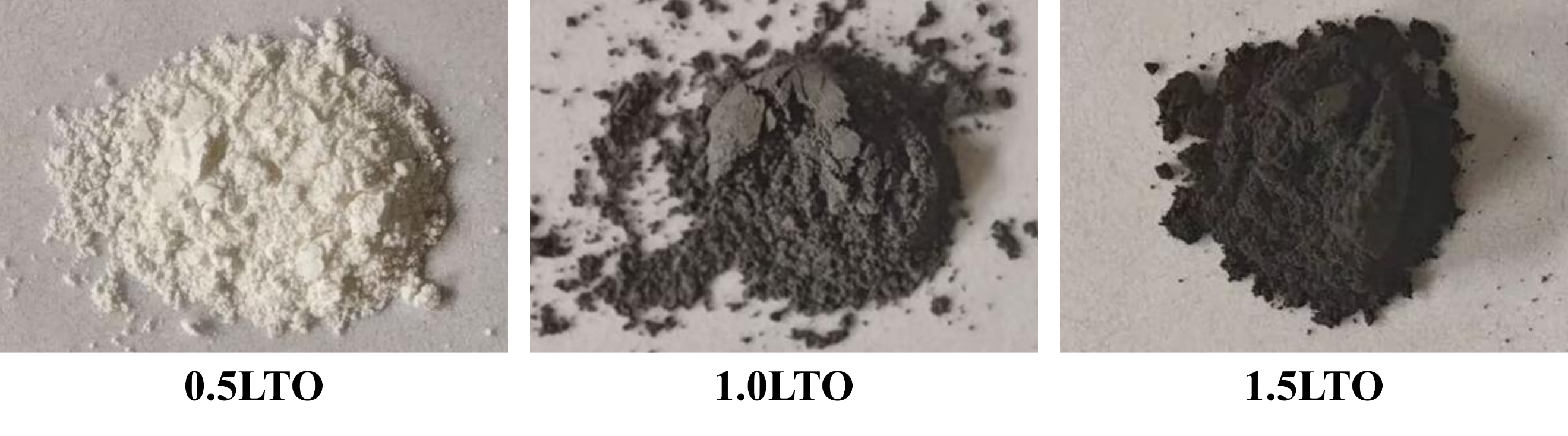


**Figure S1.** The optical image of lithium-doped TiO_2_ nanoparticles


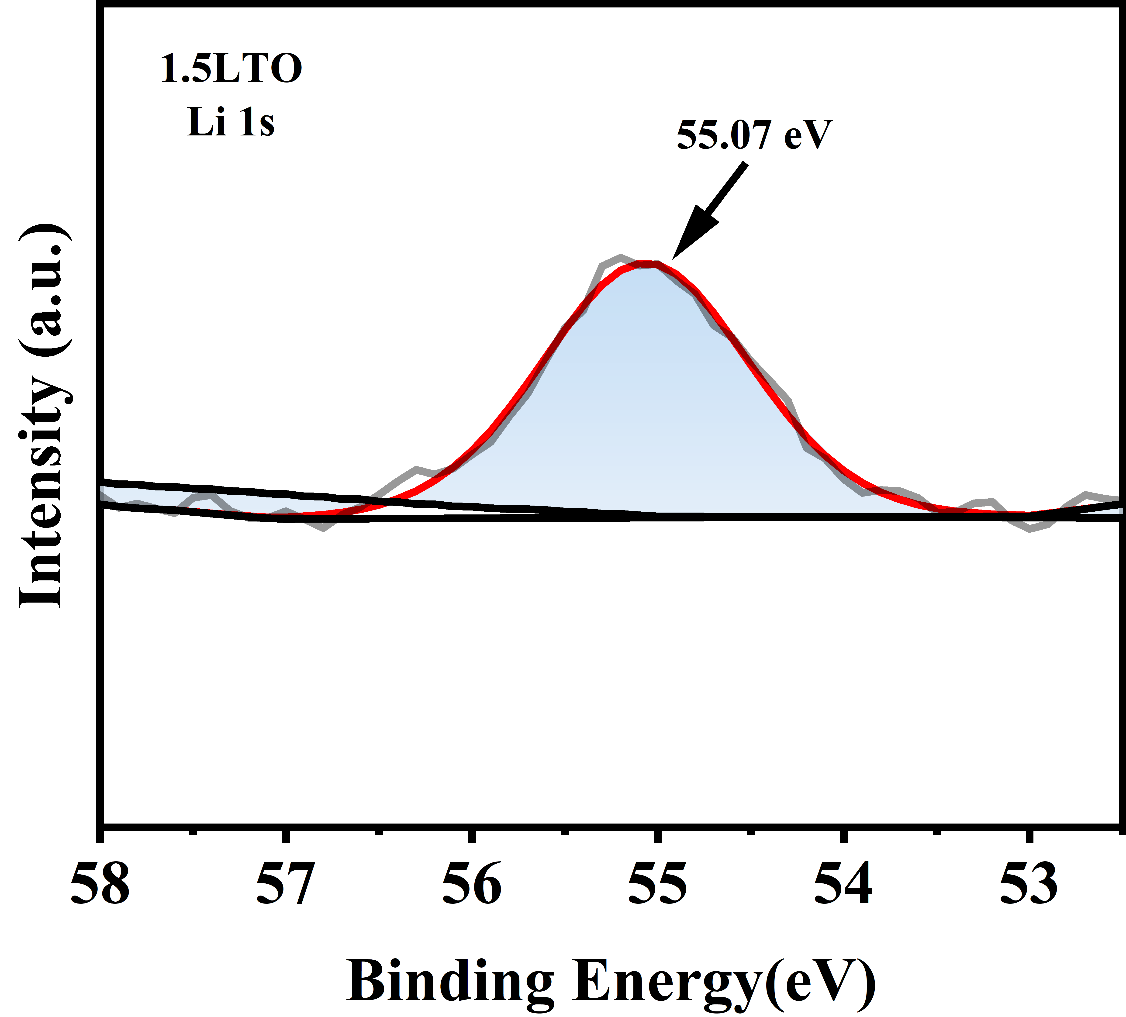


**Figure S2.** Li 1s fitting results for split peaks of 1.5LTO


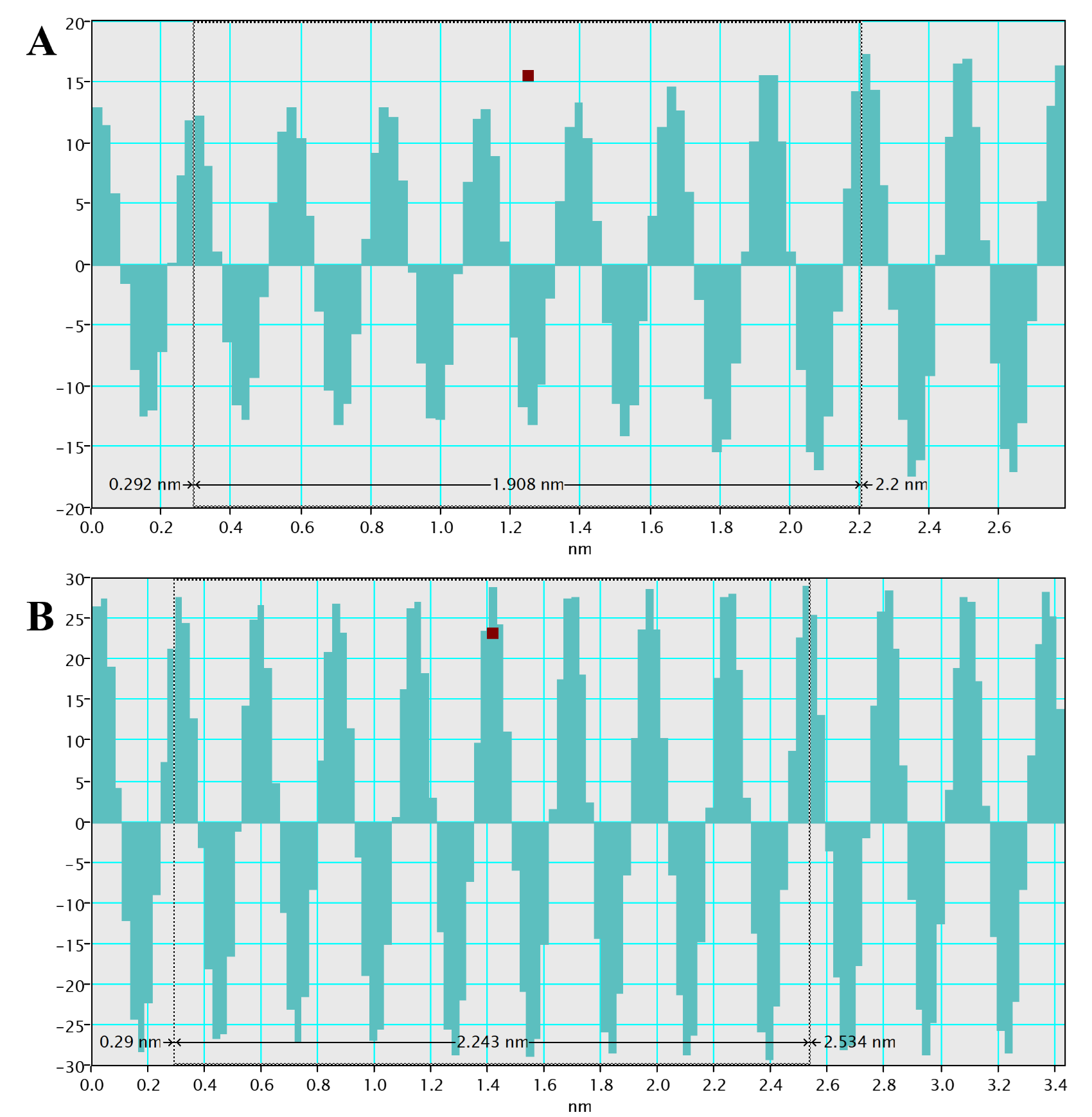


**Figure S3.** Linear measurement of lattice fringes in (A) STO and (B) 1.5LSTO nanocrystals by HRTEM


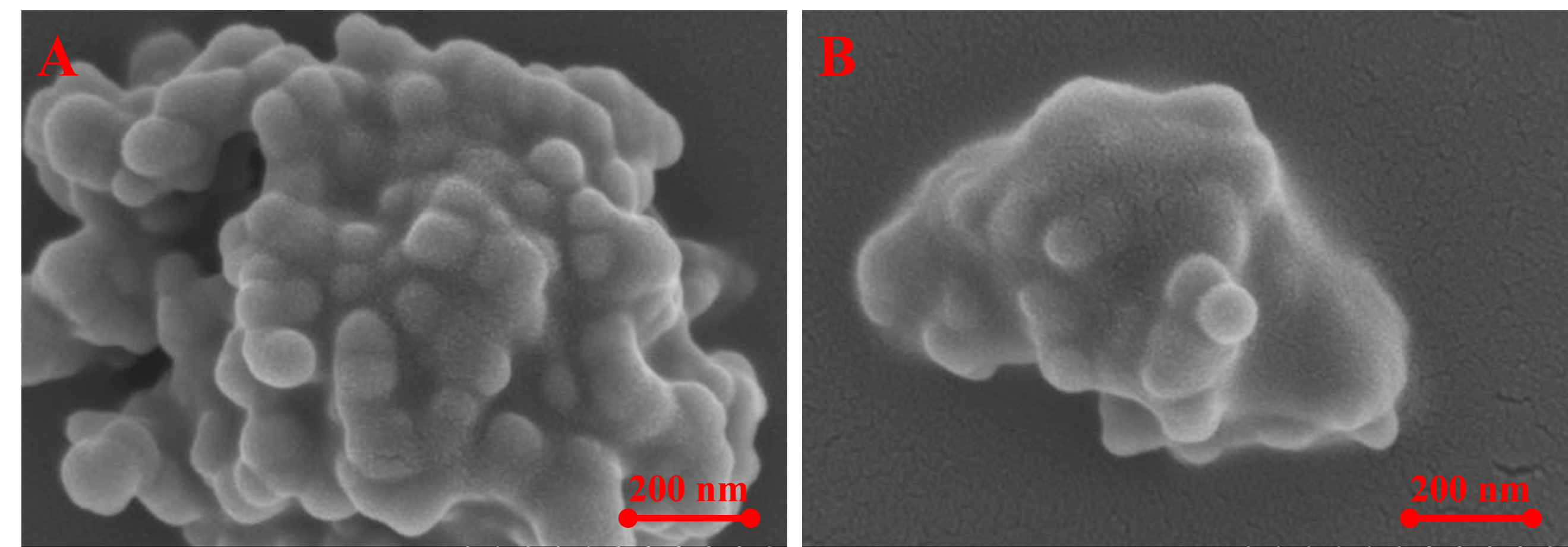


**Figure S4.** The SEM images of (A) STO; (B) 1.5LSTO

**Table S1.** The concentration of lithium ions in solution under ultrasound

| Samples | Determination of elemental concentration in solution C0（mg/L) | Elemental content of the sample（%) |
| --- | --- | --- |
| 1.5LSTO | 10.02 | 2.03% |


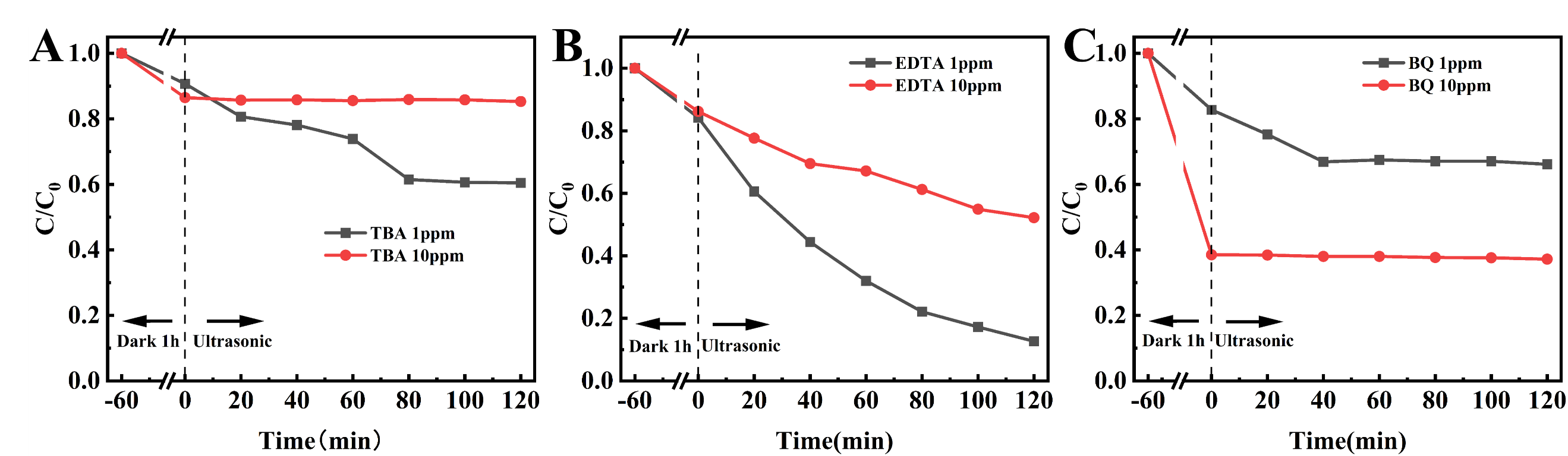


**Figure S5.** The trapping efficiency of free radicals using varying concentrations of (A) TBA, (B) EDTA, and (C) BQ


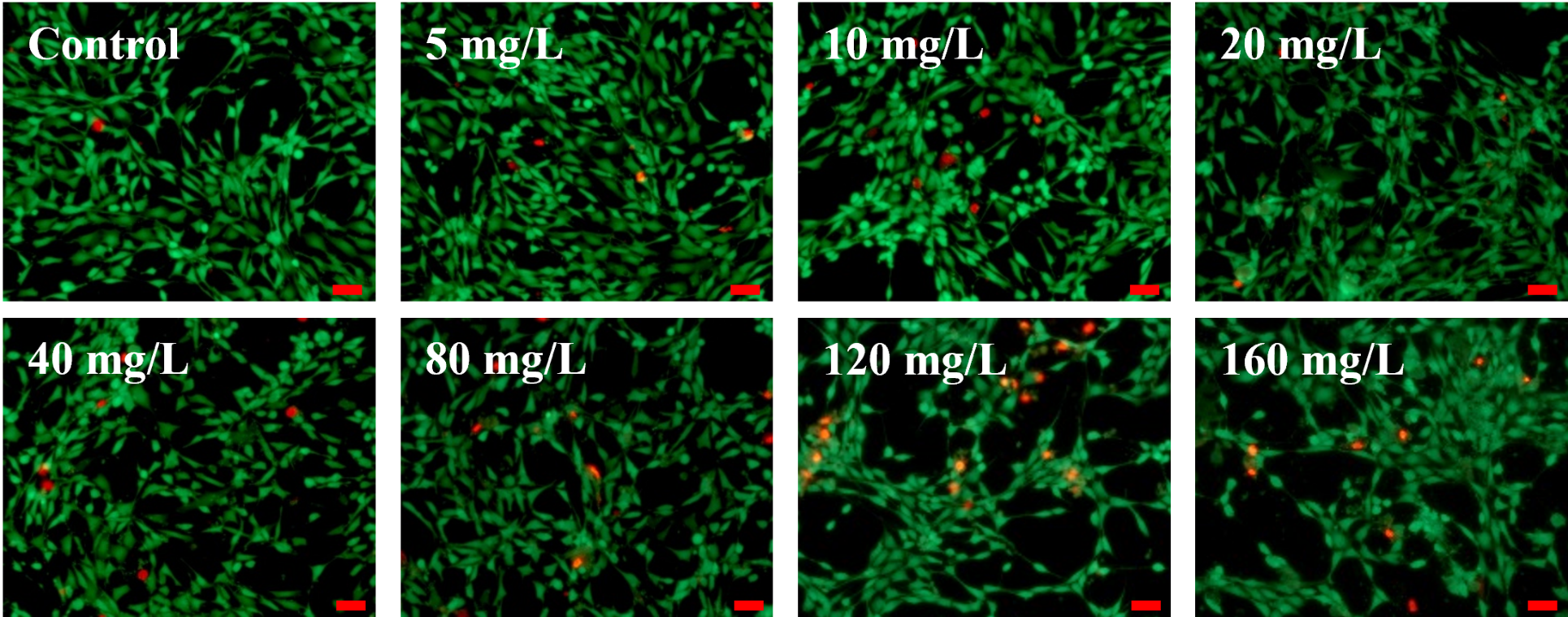


**Figure S6.** Live/dead cell staining with Calcein AM/PI of 3T3 cells incorporated with STO material. (Scale bar = 50 μm)


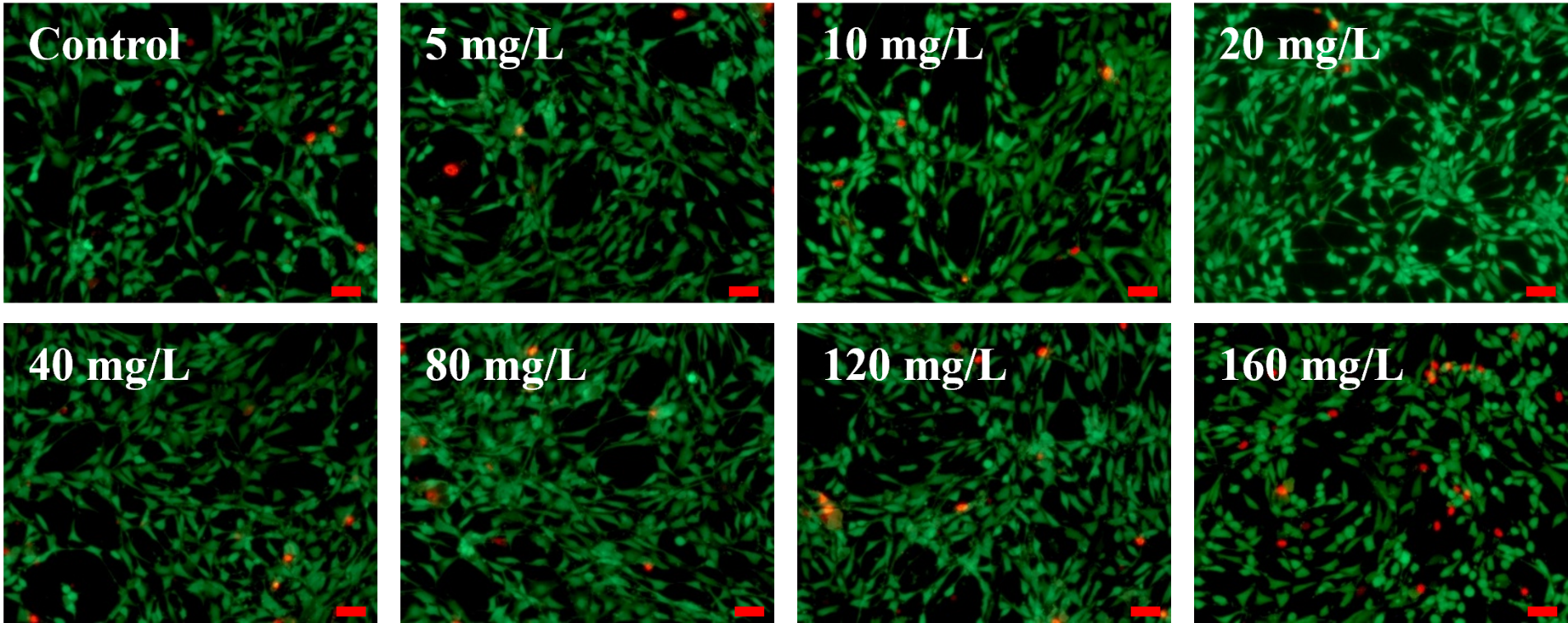


**Figure S7.** Live/dead cell staining with Calcein AM/PI of 3T3 cells incorporated with 1.5LSTO material (Scale bar = 50 μm)


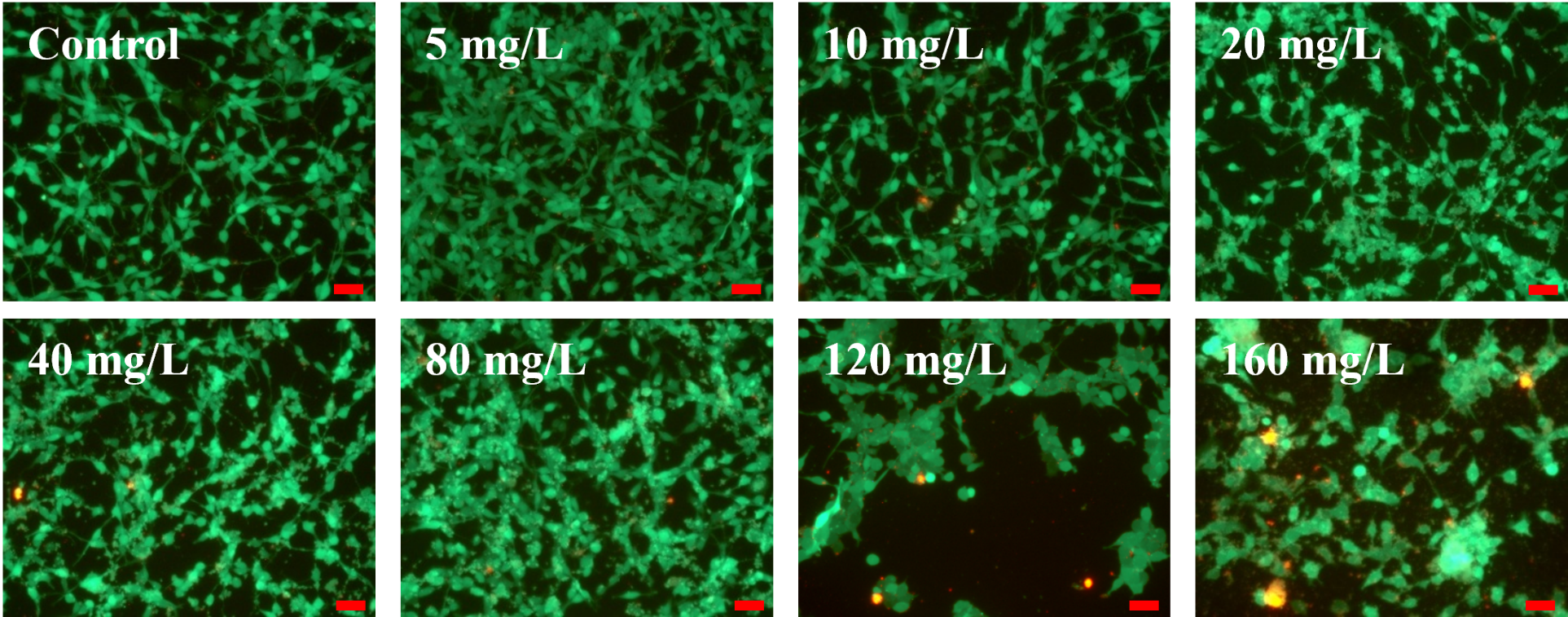


**Figure S8.** Live/dead cell staining with Calcein AM/PI of CT26 cells incorporated with STO material (Scale bar = 50 μm)


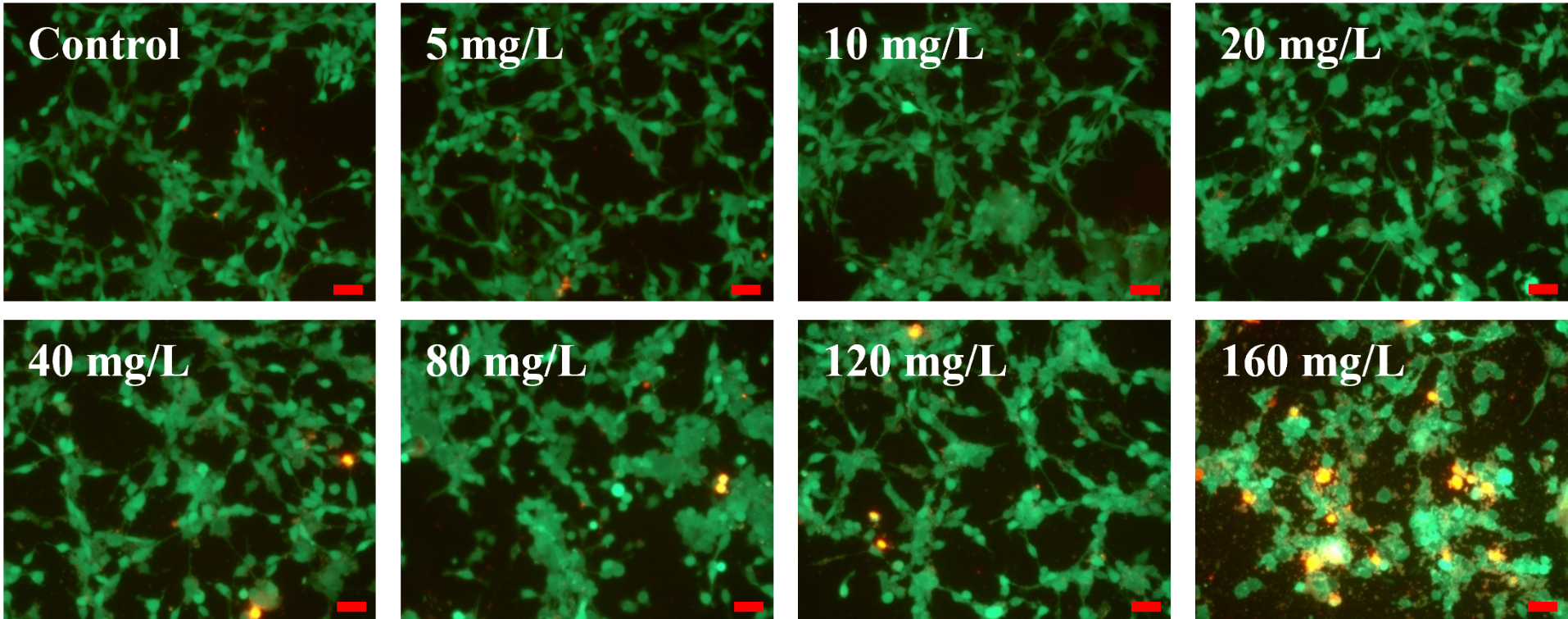


**Figure S9.** Live/dead cell staining with Calcein AM/PI of CT26 cells incorporated with 1.5LSTO material (Scale bar = 50 μm)


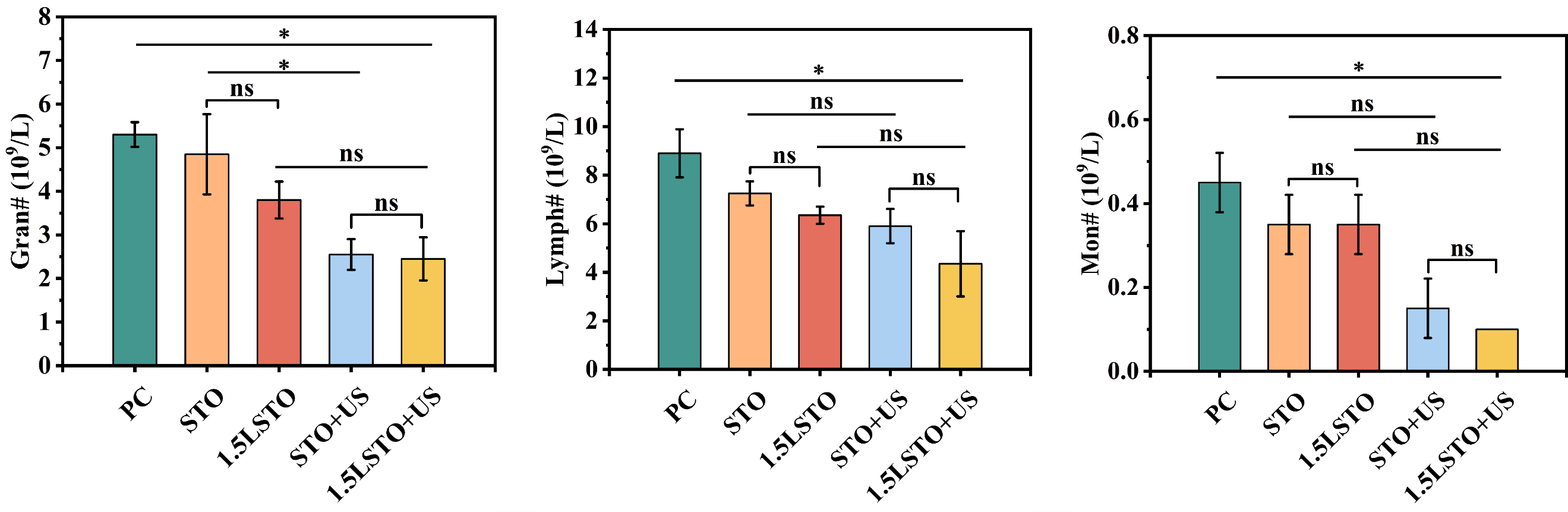


**Figure S10.** Blood biochemistry associated with inflammatory marker on day 14.


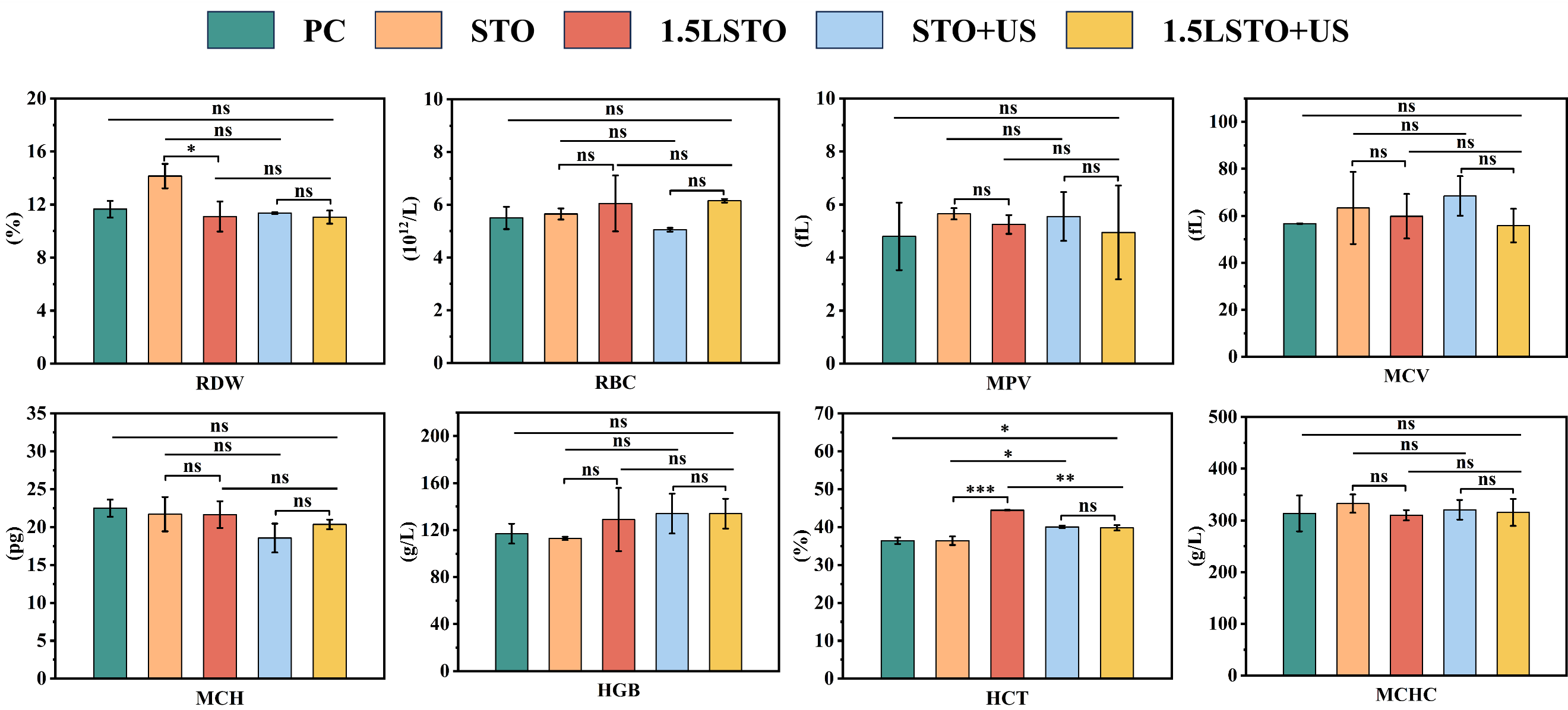


**Figure S11.** Blood biochemistry associated with non-inflammatory marker on day 14.
